# SAA4: An Underdog Within the Serum Amyloid a Superfamily?

**DOI:** 10.3390/ijms27093907

**Published:** 2026-04-28

**Authors:** Ernst Malle, Corina Madreiter-Sokolowski, Christian Windpassinger

**Affiliations:** 1 Gottfried Schatz Research Center, Division of Molecular Biology and Biochemistry, Medical University of Graz, A-8010 Graz, Austria; corina.madreiter@medunigraz.at; 2 Diagnostic and Research Center for Molecular BioMedicine, Institute of Human Genetics, Medical University of Graz, A-8010 Graz, Austria; christian.windpassinger@medunigraz.at

**Keywords:** acute-phase reactant, atherosclerosis, cancer, inflammation, glycosylation, SAA proteins, SAA4, SAA-4

## Abstract

Non-glycosylated liver-derived acute-phase amyloid A1 and A2 proteins (SAA1 and SAA2, 104 amino acids), generated by two different genes in humans (*SAA1*/*2*) and other mammalian species, are considered the prime acute-phase reactants following inflammatory conditions during host defense in cells, tissues, and the circulation. While human *SAA3* has been identified as a pseudogene, *Saa3* genes in other mammalian species are coding for primarily extrahepatically expressed Saa3 proteins that also may act as suitable inflammatory markers. The discovery of SAA4 (112 amino acids, carrying an octapeptide insert) in humans and mice has paved a new avenue for the exploration of different functions of this so far unknown member of the SAA superfamily. SAA4 has originally been termed a “constitutively” expressed SAA protein, apparently due to its nature not to act as an inflammatory marker. The present overview aimed to cover possible functions—so far identified—for human SAA4 (following its expression in various diseases on mRNA and protein level) and to work out whether SAA4 might be considered—at least in part—an acute-phase protein. Alternatively, we are raising the question whether SAA4 may solely act as a bystander or even underdog within the whole SAA family, where SAA1 and SAA2 proteins (commonly termed acute-phase SAA) hold undoubtedly an eminent status during inflammatory conditions, not only as host defense reactants but also as long-lasting markers for chronic diseases and malignancies in humans.

## 1. Introduction

The whole serum amyloid A (SAA) family is remarkably conserved during evolution, thereby providing evidence that its members may have important physiological functions, primarily in host defense and maintenance of ongoing homeostasis. All individual members of the SAA family are considered to be part of an “evolutionary old family of proteins” generated by four different genes (*SAA1*–*SAA4*) coding for corresponding four low-molecular-weight apolipoproteins (SAA1–4, 104–112 amino acids) expressed in almost all vertebrates [1,2].

### 1.1. The Acute-Phase SAA Proteins Are Primarily of Hepatic (SAA1/SAA2) or Extrahepatic (SAA3) Origin

Non-glycosylated mature SAA1 and SAA2 proteins (that differ only in 7–8 out of 104 amino acids in humans [3]) represent the major acute-phase reactants (termed A-SAA) generated primarily via cytokine-mediated induction in the liver. However, an ongoing series of publications has addressed extrahepatic A-SAA expression in humans and animals, using both primary cells and extrahepatic cell lines [4]. Besides its role as a short-term acute inflammatory marker for clinical practice, where A-SAA levels return to baseline a few days after the onset [5,6], A-SAA may further act as a potent long-term inflammatory indicator in the initiation and progression of chronic diseases such as reactive systemic amyloidosis [7], arthritis [8], atherosclerosis [9], cancer [10], chronic metabolic diseases such as obesity, diabetes, cardiovascular diseases [11] and other malignancies have been studied for more than 40 years.

The fact that human *SAA3* is a pseudogene has originally limited scientific interest in the community to reveal its potential function on the DNA and/or protein level in other mammalian species [12]. However, over the last decades, a series of fascinating findings using cell culture experiments as well as different genetic mouse models have contributed to underscore an eminent (pro/anti)atherogenic role of non-glycosylated SAA3 (primarily expressed in extrahepatic tissues) in various diseases, including adipose inflammation, atherosclerosis, hyperlipidemia, obesity, and others [13,14,15,16].

### 1.2. The Constitutively Expressed SAA Protein

Whitehead and colleagues [17] were first to publish a new member of the SAA superfamily; data confirmed later on the DNA level by Watson and colleagues [18]. As expression of this so far unknown *mRNA* in human liver tissue, with striking homology to *A-SAA* mRNA, was hardly detected following classical induction of the acute-phase response, it was designated “constitutive SAA” (C-SAA, later SAA4) [17]. Although both A-SAA and C-SAA are members of the apolipoprotein moiety of primarily lipoproteins of the high-density range (HDL) major differences between SAA1/2 and SAA4 proteins are (i) the number of amino acid residues resulting in different molecular masses, pI bands and secondary structure including hydrophilic, hydrophobic and amphipathic helical elements and (ii) the absence/presence of an octapeptide insertion optional leading to (non)glycosylated proteins in mammalian species [17,18].

The primary objective of this review was to show the genetic organization of murine *Saa4* and human *SAA4* (but not human *SAA2–SAA4*, a so-called readthrough transcript of both genes in close proximity). Next, we address candidate missense mutations in the human *SAA4* gene as well as striking protein homology between human and other mammalian SAA4 proteins. Finally, we try to figure out whether human SAA4 itself—although still considered “constitutively expressed”—might oppose this prediction in clinical studies not directly focusing on malignancies or even acting as a likely biomarker in various human cancer types. Finally, we were interested in whether any convincing line of evidence exists that human SAA4 might be associated with any inherited disease.

## 2. Ideogram and Location and Genomic Organization of SAA4/Saa4 on Respective Chromosomes

**Figure 1:**
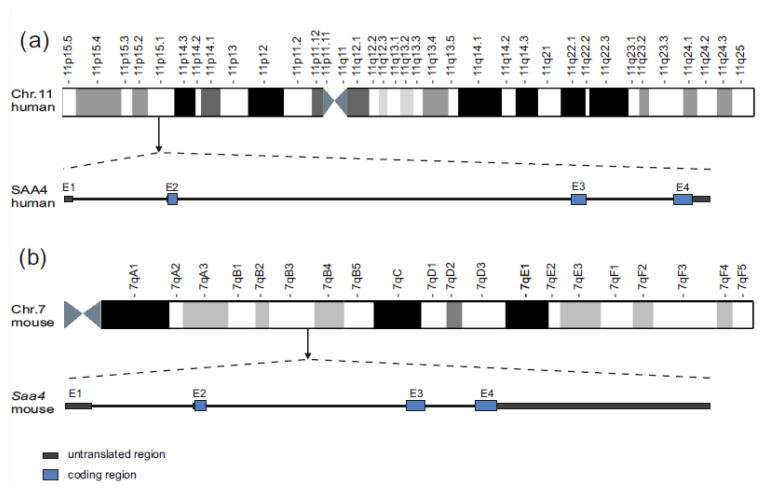
Comparison of (**a**) human SAA4 and (**b**) murine Saa4 gene organization: All graphical and numerical information included in the figure is depicted and adapted from the UCSC genome browser, human genome (hg38), and mouse genome (mm39) assembly. Ideograms are adapted from Wikimedia Commons content on a data basis. In general, the exon/intron structures of both the human *SAA4* (NM_006512.49) and the murine *Saa4* (NM_011316.4) genes show untranslated regions (dark gray rectangles), translated regions (blue rectangles), and intronic regions (black lines).

The human *SAA4* gene is localized on chromosome 11 within 11p15.1, while the murine *Saa4* gene is localized on chromosome 7 within 7qB3 (Figure 1a). Basically, the human *SAA4* RefSeq mRNA (NM_006512.4, NCBI) consists of one 5′UTR untranslated region (exon 1) and three coding exons (exon 2–4) and spans a genomic region of 5448 bp on chromosome 11 from 18,231,355 base pairs (bp) to 18,236,802 bp; its coding orientation is on the reverse strand.

The murine *Saa4* RefSeq mRNA (NM_011316.4, NCBI) also consists of one 5′UTR exon (exon 1) and three coding exons (exon 2–4) and spans a genomic region of 4606 bp on chromosome 7 from position chr11: chr7:46,727,998–46,732,603; again, its coding orientation is on the reversed strand (Figure 1b). A more detailed description of the murine *Saa4* gene, as well as its linkage to other members of the SAA gene family, has previously been published by several groups [19,20,21,22].

## 3. Sequence Comparison of Selected Mammalian (SAA4/Saa4) Proteins by the ClustalW Alignment

Following the whole recently reconstructed evolutionary tree of all known SAA clades, a panel of different species, such as bacterial, brachiopod, bird, cephalochordate, fish, and mammalian clades (including mammalian clades SAA1–SAA4), as well as marine echinoderm, mollusc, oyster, spider, tick, turtle and crocodile clades, has been published [23]. The mammalian SAA4 clade (containing ten species) served as the basis for the present protein alignments. Seven representative organisms (all of them with identical lengths of 130 amino acids) were chosen for sequence comparison, whereas others, such as *Cavia porcellus* (guinea pig, 127 amino acids), *Oryctolagus cuniculus* (European rabbit, 135 amino acids), and *Pongo abelii* (Sumatran orangutan, 110 amino acids) were excluded.

**Figure 2:**
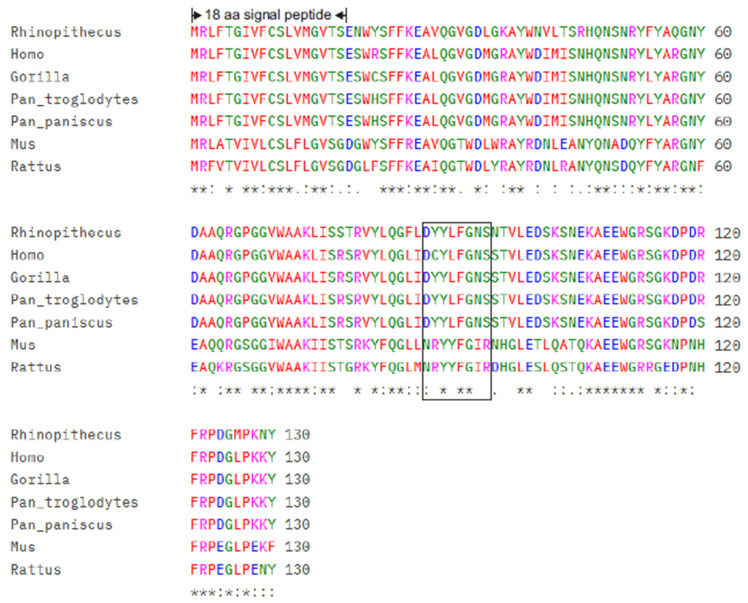
ClustalW multiple sequence alignment of SAA4 proteins: All sequences are aligned in rows, broken into 60-character blocks, with respective species names on the left. ClustalW alignment [24] of SAA4 orthologs from the indicated mammalian species illustrates strong conservation within the N-terminal 18 amino acids leader/signal peptide of pre-SAA4 (130 amino acids) of the following species are displayed: *Rhinopithecus* (black-and-white snub-nosed monkey), *Homo* (human), *Gorilla* (gorilla), *Pan troglodytes* (chimpanzee), *Pan paniscus* (bonobo), *Mus* (mouse), and *Rattus* (rat). Conserved residues within mature SAA4 proteins (112 amino acids) address regions under strong evolutionary homology, while variable positions are limited and largely conserved. (no symbol) = positions with no conservation; (*) = positions with a single or fully conserved residue (identical); (**·**) = conservation between groups of weakly similar properties (scoring > 0 and ≤0.5 in the accepted point mutations (PAM 250 matrix); (:) = conservation between groups of strongly similar properties (scoring > 0.5 in PAM 250 matrix). The rectangle starting after position 69 of the indicated mature SAA4 species marks the octapeptide insertion.

Figure 2 shows seven different pre-SAA4 protein sequences (130 amino acids). The respective leader or signal peptides (18 amino acids) will be cleaved off from respective pre-SAA4-proteins from all species, resulting in secretion and often direct association of mature C-SAA (SAA4) proteins (112 amino acids) with HDL subfraction 3 (HDL3). The percentage homology between species is addressed in Table 1.

Figure 2 further shows the polypeptide insertion after position 69 (referring to amino acid position of 70–77) within the mature SAA4 protein (112 amino acids). The potential glycosylation site within this octapeptide starts at position 7 (arginine, N) with a NSS (Arg-Ser-Ser) motif, present in humans, *Pan troglodytes* (common chimpanzee), *Pan paniscus* (bonobo) and *Gorilla* (gorilla) but not in other mammalian species. However, an evolutionary paradox is the observation that in some A-SAA proteins, e.g., horse (perissodactyls), dog and mink (carnivores), an octapeptide is similarly positioned (see above), whereas in cow and sheep (artiodactyls), even a nonapeptide was found [2,21,25,26,27].

Previous studies revealed that commercially available recombinant human SAA1 (carrying an N-terminal Met) chemoattracts monocytes to increase cytoplasmic Ca^2+^ levels that activate non-pancreatic phospholipase A_2_ to cleave the fatty acid moiety at the *sn*-2 position of phospholipids to be further metabolized to eicosanoids [28,29]. While the highly conserved tetrapeptide G^48^-P^49^-G^50^-G^51^ in SAA1/2 and SAA4 (see Figure 2), being homologous to G^30^-X^31^-G^32^-G^33^ (the main lipid and Ca^2+^ binding site on phospholipase A_2_ molecules), is not responsible for A-SAA-mediated eicosanoid biosynthesis of preactivated monocytes [30] it might be worth revealing whether this tetrapeptide, also present in human and monkey SAA4 proteins, might bind Ca^2+^ ions to further promote phospholipase activity for biosynthesis of prostaglandins and/or thromboxane.

Immunoelectrophoresis technique revealed that the N-terminal arginine of A-SAA may easily be cleaved off by various enzymes, leading to the 103 amino acid truncated des-Arg form of SAA1 and SAA2 [31,32]. MALDI-TOF mass spectrometry and *m*/*z* calculation of the delipidated human HDL-associated apolipoprotein moiety revealed—in contrast to N-terminal truncation of SAA1/2—truncations for SAA4 at the C-terminal domain, leading to the loss of either two (Lys-Tyr, 110 amino acids) or three amino acids (Lys-Lys-Tyr, 109 amino acids) [33].

## 4. DNA Variants in SAA4 from gnomAD

To assess the spectrum of protein-altering variations in human SAA4, data were extracted from the Genome Aggregation Database (gnomAD v4.1.0). In total, 217 distinct variants, potentially affecting the amino acid sequence of SAA4, were found in the coding and splice site regions of *SAA4* in the data set of approximately 1.6 million alleles analyzed. From these alterations (n = 217), the majority were “missense” variants (n = 192), followed by “splice site” variants (n = 7), “frame shift” variants (n = 6), “stop gained” variants (n = 5), “start lost” variants (n = 3), “stop lost” variants (n = 2), as well as “splice donor” variant (n = 1) and “in-frame deletion” variant (n = 1). The 6 “frameshift variants” consist of four “deletions”, one “insertion”, and one “duplication”.

One of the naturally occurring 192 missense variants of human SAA4 is the common missense polymorphism p.Cys89Tyr (rs2460827). Although the human reference sequence lists Cys at this position, large population datasets such as gnomAD demonstrate that Tyr represents the major allele (with approx. 1.54 million alleles analyzed, Gnomad v.4.1.0).

Table 2 further shows the total allele frequency of rs2460827, ranging from 73.8% in African/Americans to 100% in Amish people, with an overall frequency of approximately 96%.

## 5. Association of SAA4 with Lipoproteins

Mean serum SAA4 levels estimated by ELISA (using recombinant SAA4 and a monoclonal anti-SAA4 antibody) were 115 mg/dL (ranging between 80 and 140 mg/dL) in normal subjects [34,35] or 55 ± 13 μg/mL (mean plasma concentrations) using an immunoradiometric method and a polyclonal rabbit anti-human SAA4 antibody [36]. While minor amounts of SAA4 were identified in both VLDL [37] and LDL plasma lipoproteins [38,39], the majority was present in HDL subfractions (HDL_2_ and HDL_3_ [40,41] but not in pre*β*-HDL particles [42]; data finally confirming SAA4 as a real component of the whole apolipoprotein moiety in humans and mice [43]. However, whether the presence of SAA4 within the HDL proteome might act as a mediator or definitive marker for artery disease was originally not clear [44]; however, ongoing studies revealed that, in contrast to SAA1 protein levels, SAA2 and SAA4 were significantly decreased in coronary artery disease patients compared to controls [45]. Both A-SAA and SAA4 were detected in human lesion material, and SAA4 was also found to be associated with LDL particles in hypertriglyceridemia [46].

## 6. N-Glycosylation of SAA4

Glycosylation of proteins occurs in the endoplasmic reticulum and the Golgi apparatus, but also in the nucleus and cytoplasm [47] and may alter protein folding, transport, stability, and assembly in health and disease [48]. This potential post-translational modification via macromolecules called glycans may occur via *O*-linkage (attached to a hydroxyl group) or *N*-linkage (attached to Asn residues) with a consensus sequence of *N-X-T/S*; *X* may be any amino acid except Pro, and the position of *X* might be a determining factor for the extent of *N*-glycosylation [49]. Several groups using different methodological approaches confirmed the presence of a 14 kDa and 19 kDa (glycosylated) SAA4 protein [17,32,33,50]; the ratio between both (un)glycosylated forms is basically considered approx. 1:1 [17]). The first study (using two different constructs called SAA4^WT^ and SAA4^N76Q^) revealed that disruption of glycans in SAA4^N76Q^ did neither modulate secretion nor degradation of SAA4; however, glycosylation of SAA4 may alter its distribution within different lipoprotein particles [51]. Indeed, glycosylation of small molecular mass apolipoproteins may be associated with lipoprotein-mediated and/or initiated diseases [51]. Savinova and colleagues [52] reported a decrease in glycosylated apo C-III (17% in VLDL; 30% in LDL, 25% in HDL), apo E (15% in VLDL, 26% in LDL, 37% in HDL), and apo SAA4 (42% in HDL) in patients with metabolic syndrome. Yamada and colleagues revealed that SAA4 variants (position 71: Tyr/Tyr or Tyr/Cys) or (position 84: Ser/Ser or Ser/Leu) might alter the extent of *N*-glycosylation but not total plasma SAA4 levels [53]. In a case–control study investigating HDL-associated apolipoproteins in patients with neuropathic pain, six SAA4 isoforms could be identified (similar to that reported previously by Karlsson and colleagues [50]), while only two SAA4 isoproteins could be detected in control HDL [54].

A strong association between chronic periodontitis and coronary heart disease is paralleled by alterations in both the lipid and protein moieties of the respective lipoproteins [55,56]. The redistribution of the apolipoprotein moiety in lipoprotein particles from periodontitis patients reveals an altered balance between glycosylated and non-glycosylated SAA4, but decreased expression of at least one glycosylated form [56]; findings that parallel previous data obtained when blood was stimulated ex vivo with *Porphyromonas gingivalis* [57]. Observations that SAA4 could even serve as an optional marker (beyond its presence in laboratory and clinical medicine) in dentistry are supported by its role during in vivo osseointegration using either regular Titan implant surfaces or surfaces preabsorbed with Ca^2+^ ions [58,59].

## 7. Previous Data on Expression of SAA4 on mRNA and Protein Levels in Cells, Cell Lines, and Tissues

### 7.1. Hepatic Expression of SAA4

A sequenced cDNA clone isolated from a human liver library was considered the basis to predict an SAA isoform of 130 amino acids, where the leader peptide of 18 amino acids has been cleaved off [17]. As human acute-phase liver contained only small amounts of this new “*SAA* mRNA” (compared to A-*SAA* mRNA), and the fact that treatment of human PLC/PRF/5 hepatoma cells with cytokine-containing monocyte-conditioned medium also did not induce mRNA levels of this new “*SAA* mRNA” (compared with *A*-*SAA* mRNA), led to the terminology of a constitutive character of this new SAA protein [17]. While PCR analysis revealed only a single band present in the human liver [60], Northern blots showed two bands (upper and slightly lower) in various human hepatoma cell lines (Hep3B, different HepG2 cell clones, as well as PLC/PRF/5 and HuH7 cells), depending on the absence/presence of dexamethasone or monocyte-conditioned medium [61,62]. Possible explanations for both bands were (i) the use of two different transcription start or polyadenylation sites, (ii) alternative splicing of the primary transcript, or (iii) the differential polyadenylation of the same transcript [61].

The study by Wunderlich and colleagues aimed at time-dependent expression of hepatic genes (encoding proteins involved in coagulation/fibrinolysis, complement regulatory proteins, and others) with *Plasmodium chabaudi* or protective vaccination of Balb/c mice prior to malaria infection. When mice recovered due to protective vaccination, four genes encoding for acute-phase proteins such as *serum amyloid P component*, *caeruloplasmin*, *C-reactive protein*, and *Saa4*, were found on day eleven after treatment [63].

### 7.2. Extrahepatic Expression of SAA4

The first experimental setting using PCR revealed *SAA4* mRNA expression in cultured myeloid 1α,25-dihydroxyvitamin (D_3_)-treated THP-1 cells (a human leukemia monocytic cell line) stimulated with lipopolysaccharide and dexamethasone [60] as well as in cultured aortic smooth muscle cells in response to dexamethasone/IL-1 or IL-6 [64]. *SAA4* mRNA was also detected in cultured human aortic smooth muscle cells, but expression levels remained at quiescent levels [65]. While A-SAA was found to be chemotactic for human aortic smooth muscle cells, SAA4 did not affect cell migration [66]. In line with these findings, Liang and colleagues further reported that, in particular, the N-terminal region of A-SAA may be responsible for binding and transferring cholesterol to both smooth muscle and HepG2 cells, an observation that is not surprising and dependent on the different primary and secondary structures of both SAA1 and SAA4 [67].

Expression of *SAA4* mRNA was also confirmed in human monocytes (in the absence/presence of dexamethasone or lipopolysaccharide), non-adherent and polarized monocytes, as well as cultured macrophages [62]. While in human coronary arterial endothelial cells, cytokine- and/or dexamethasone-mediated mRNA expression was only found for *SAA1/2* but not *SAA4*, a similar intracellular localization, predominantly in the perinuclear region and the nuclear space, was found for all three SAA proteins [68].

Northern blot experiments in MRC5 (fetal lung fibroblasts) and ECV304 cells (umbilical cord endothelial cells) revealed only a single band for *SAA4* mRNA (co-migrating with the upper band found in hepatoma cells, see above) that irrespective of different treatment conditions remained unaltered even during time course experiments (up to 24 h) when cells were treated with actinomycin D before and after cytokine (IL-1 and IL-6) treatment [61].

Following expression of *SAA4* on mRNA level by RT-PCR in human bone tissue (trabecular bone, cortical bone and bone marrow), as well as in non-differentiated and differentiated (so-called primary osteoblasts) human mesenchymal stem cells treated with cytokines (IL-1α, IL-1β, TNFα or IL-6), *SAA4* mRNA was solely constitutively expressed, although respective cytokine receptors were present in these biological specimens [69]. Similar findings were also obtained in rheumatoid synovial fibroblasts when treated with IL-18 [70]. IL-6 trans-signaling promoted expression of *A-SAA* mRNA and subsequent activation of the JAK2/STAT3 pathway (an inflammatory pathway completely abrogated by CP690,550) in human rheumatoid arthritis fibroblast-like synoviocytes; nevertheless, *SAA4* mRNA remained constitutively expressed even when treated with IL-6, sIL-6R, or IL-6/sIL-6R [71].

In terms of placental tissue, i.e., decidua and villous trophoblasts, constitutive expression of different *SAA* mRNAs was decreased in the following order: *SAA1* >> *SAA2* > *SAA4* [72]. Low expression of *SAA4* transcripts in early first trimester pregnancy weeks (7 to 9), pronounced expression in weeks 10 and 12 [73], expression of *SAA4* during natural cycle frozen embryo transfer, as well as in pregnancies achieved by means of cryopreserved embryos under unassisted conceptions [74] and finally constitutive *SAA4* expression in outgrowing extravillous trophoblasts [73], suggested a potential role of SAA4 during trophoblast invasion, maybe to counteract the development of preeclampsia [74]. However, expression of *SAA1/2* as well as *SAA4* mRNA was also confirmed by RT-PCR in granulosa cells recovered from follicular aspirates of in vitro fertilization patients [75].

While A-SAA expression could also be confirmed by in situ hybridization and immunohistochemistry in a broad panel of normal human tissues, RT-PCR analyses of basal *SAA4* mRNA expression were only positive in spleen, large intestine, kidney, esophagus, and breast [76]. Most importantly, breast-derived *SAA4* sequence data used in this study had a nucleotide homology of 99% with clone M81349 originally used by Whitehead and colleagues [17]. This homology not only acts as definitive proof to confirm findings but also for data obtained for colonic tumorigenesis, where RT-PCR analyses clearly revealed similar expression of *SAA1* and *SAA4*, respectively [77,78].

Relative expression of *SAA4* mRNA in different cell types from surgical lung samples from a cohort of chronic obstructive pulmonary disease patients (n = 40) was quite similar as found for *SAA2* mRNA in lung fibroblasts, while only basal expression was found in lung epithelial cells and lung macrophages; unfortunately, observed gene expression was not related to any tumor type, corticosteroid intake or degree of lung function [79]. In another cohort of 40 chronic obstructive pulmonary disease patients (eleven in disease stage I, 24 in stage II, and five in stage III), expression of all three human SAA transcripts was individually increased in lung parenchyma compared to pulmonary arteries as well as in blood leukocytes; most importantly, *SAA4* mRNA expression in parenchyma and blood was highest in stage III [80]. Another study reported a similar increase in *SAA1/2* (4.36/3.65-fold) and *SAA4* mRNA (3.9-fold) in lung bronchial chronic obstructive pulmonary disease tissue, while expression of SAA4 (in contrast to SAA1/2 and C-reactive protein) in parenchymal lung tissue was similar compared to controls [81].

While the acute-phase response (i.e., a dramatic expression of A-SAA) could be confirmed by immunoblot analysis in brain tissue from patients with Alzheimer’s disease but not in patients with Lewy Body or Pick’s disease, SAA4 could not be verified previously in any brain tissue samples [82]. Most importantly, expression of *Saa1* mRNA levels in murine brain following intracerebral inoculation with a strain of Sindbis virus (mimicking inflammatory conditions) revealed remarkable differences between liver- and brain-derived *Saa1* expression [83].

High throughput RNA sequencing of differentially expressed genes in gingival tissue from 10 chronic periodontitis-affected patients (compared with 10 healthy gingival tissue specimens) revealed 62 genes downregulated while the top upregulated genes (out of 400) included cytokines and immune related genes (colony stimulating factor 3; chemokine (C-X-C motif) ligand 1 (melanoma growth stimulating activity, alpha; lipopolysaccharide binding protein), proteases (matrix metallopeptidase 3 and 7; membrane metallo-endopeptidase; membrane metallo-endopeptidase antisense RNA 1) and all three members of the human SAA family (*SAA1*, *SAA2*, and *SAA4*) [84].

### 7.3. Expression of SAA4 in Cancer Cells

While the adverse role of A-SAA, its association and underlying mechanisms in the pathogenesis of cancer have been extensively studied for decades [for review see: [10,85,86,87]; scarcely any data are available for SAA4. Northern blot experiments confirmed the constitutive character of human *SAA4* mRNA in cell lines such as SW13 (an adrenal cortex carcinoma), RT4/31 (a bladder papilloma), HCT-8 (an ileocecal carcinoma), KB (an oral epidermal carcinoma), Hela Ohio (a cervical carcinoma), CaCo2 (a colon adenocarcinoma) and U937 (a histocytic lymphoma) irrespective cellular treatment with monocyte-conditioned medium, dexamethasone or both agonists [61].

Also, in human osteoblast-like cell lines of tumor origin (MG-63 and SAOS-2, both of them expressing a panel of prominent cytokine receptors), *SAA4*, in contrast to *SAA1/2* mRNA, remained constitutively expressed even after cytokine treatment with IL-1α, IL-1β, TNFα or IL-6 [69].

Basal expression of different *SAA* mRNAs in human BeWo choriocarcinoma cell line decreased in the following order (*SAA1* >> *SAA2* > *SAA4* [72]. Both RT-PCR and qRT-PCR techniques performed in human trophoblast-like choriocarcinoma cell lines Jeg-3 and AC1-M59 (a hybrid of term trophoblast cells and AC-1 cells derived from Jeg-3) revealed the presence of *SAA4* mRNA, which, however, was not responsive to IL-1β, IL-6 and TNFα but significantly downregulated by IL-1α [73].

Ren and coworkers [88] focused on the expression of SAA transcripts in uterine cervical cancer (138 serum specimens and 31 tissue samples). RT-PCR expression of *SAA1* and *SAA4* in cervical carcinoma tissue (compared with non-neoplastic lesions) and qRT-PCR underscored their potential as optional biomarkers.

*TWIST*, a prominent oncogene upregulated in a variety of human carcinomas, might even act as a prognostic biomarker for prostate cancer. Indeed, identification of ten genes supposed to act as prime targets for *TWIST*-overexpression in malignant prostate cancer includes, among other also, *SAA4* [89].

## 8. SAA4: A Potential Tumor-, Bio- or Surrogate-Marker? Data from Clinical Studies?

### 8.1. SAA4: A Potent Tumor Marker?

Plasma-derived extracellular vesicles are considered a potent source of proteins, which, when subjected to proteomic analysis, might reveal candidate biomarker(s) to further unveil mechanisms that could be of potential diagnostic and subsequent prognostic interest in both initiation and/or progression of vascular injury and malignancies. Following expression of proteins from large extracellular vesicles in diabetic mice, pro-α1 chains of type III collagen, hair patches, and thrombospondin-4 were upregulated, while ankyrin-1 and Saa4 were downregulated [90]. At least in small extracellular vesicles isolated from multiple myeloma patients compared to patients suffering from monoclonal gammopathy, all three human SAA isoforms (A-SAA and SAA4) were highly upregulated [91]. Identification of extracellular vesicle-derived proteins revealed seven candidates (chromosome 20 open reading frame 3, tetranectin, inter-alpha (globulin) inhibitor H4, serpin peptidase inhibitor clade F, serpin peptidase inhibitor clade C, SAA4 and CD5 antigen-like); however, only the latter one (with the highest AUC value of 0.943) has been suggested as a potential extracellular vesicle biomarker for liquid biopsy in lung cancer [92]. Although expression of SAA1 (7 out of 31 peaks) has been considered the prime indicator in plasma of non-small cell lung cancer patients with poor outcome following epidermal growth factor-tyrosine kinase inhibitor treatment, SAA4 (3 peaks each out of 31) identified by differently expressed VeriStrat good and VeriStrat poor classified patients in a clinical trial, is ranking on a similar level as found for SAA2 [93]. The Veristrat assay, a combination of proteomic approach and MS, is basically considered and thus adapted for cancer treatment, including immune checkpoint inhibitor therapy (using plasma or serum as biological specimens), revealing beta-2 microglobulin, C-reactive protein, SAA1, SAA2, and SAA4, respectively, in non-small cell lung cancer patients who are most likely real responders to therapy [94].

Bladder cancer, the most common urinary tract carcinoma, is known for its high recurrence tumor rate, implying that early diagnosis and prediction of respective tumor stage(s) from urine specimens is an outstanding challenge. Following depletion of abundant urine proteins, iTRAQ labeling, LC-ESI MS/MS analysis as well as protein identification and quantification by sequence database search, detection of six apolipoproteins (apo A-I, apo A-II, apo A-IV, apo B, apo E and apo M) was followed by three additional proteins (apo A-IV, triosephosphate isomerase and SAA4) highly upregulated while pro-epidermal growth factor was remarkable decreased in all bladder subgroups thus supporting the notion that the binary combinational element of SAA and pro-epidermal growth factor may act as useful biomarkers. These findings were supported by the fact that urine SAA4 levels were gradually increasing within stages of bladder cancer, from non-invasive papillary carcinoma, low grade with early stage (n = 30), high grade with early stage (n = 50), and high grade with advanced stage (n = 25). Estimation of both biomarkers in urine revealed that levels of SAA4 are also statistically increased in both renal and transitional cell carcinoma, while pro-epidermal growth factor (measured in parallel) was not significantly decreased in urine from kidney cancer patients [95].

In search for candidate biomarkers for hepatocellular carcinoma (with a better predictive efficacy than alpha-fetoprotein), gene set enrichment analysis, gene ontology and receiver operating curves revealed that (i) SAA4 expression and immunohistochemical staining was lower in patient (n = 375) versus control (n = 50) tissues, (ii) SAA4 expression in stage I and II, G1+2 and T1+2 were higher compared to stage III and IV, G3+4 and T3+4 and that (iii) a five year overall survival analysis within the low SAA4 expression group was significantly worse compared to the high SAA4 expression group [96]. A pilot (but clinically highly relevant) proteomic exploration for comparative analysis of intrahepatic cholangiocarcinoma and other adverse liver conditions revealed, after MS-based data and ELISA validation, four proteins (leucin-rich alpha-2-glycoprotein, vascular cell adhesion protein 1, SAA1, and SAA4) that underscore their capacity as biomarkers to distinguish intrahepatic cholangiocarcinoma from primary sclerosing cholangitis and hepatocellular carcinoma [97].

Pronounced expression of *SAA4* (besides *SAA1*) mRNA in OVCAR-3 (an ovarian cancer cell line) might be considered a possible hint for SAA4 to act as a candidate cancer marker [98]. Similarly, as mentioned above, *SAA1* and *SAA4*, but not *SAA2* mRNA, were highly expressed in OVCAR-3 cells and in ovarian tumors, but not in adjacent tissues; a possible hint that both SAA1 and SAA4 might be involved in proliferation, potentially modulating the cell cycle [99]. However, to focus on suitable tumor biomarkers in epithelial ovarian cancer, ovarian cyst fluids seem to act as the most promising biological specimen for diagnosis and optional prediction of therapy. As protein levels of potential markers are apparently higher than in circulation, a more specific differentiation between benign or malignant tumors (including different stages of malignancy) might be possible. SAA4 is ranking significantly (*p* < 0.0001; ROC AUC values of >0.70) among 17 proteins (out of 1180 molecular mass protein peaks) to differentiate between malignant and benign cyst fluid samples [100]; data further supported by findings in 68 ovarian cyst fluid samples with mixed histology, where SAA4 protein concentrations increased from stage I/II (benign) to stage III/IV (malignant), although plasma SAA4 concentrations (estimated by iTRAQ-MS) did not significantly differ between malignant and benign stages [100]. Using the label-free quantification proteomics technique to identify novel biomarkers for endometrial cancer in 36 patients, subsequent validation by immunoblot analysis revealed upregulation of eight proteins (apo A-I, hemoglobin subunit beta, apo (a), SAA4, apo E, carbonic anhydrase 1, hemoglobin subunit delta and platelet factor 4 variant), while only the latter four proteins might be considered useful biomarkers for clinical stages [101]. While plasma levels of C-reactive protein were significantly increased in histologic chorioamnionitis patients with preterm premature membrane rupture compared to non-histologic chorioamnionitis patients, no difference was found for complement C4-A, transforming growth factor beta, and SAA4 [102].

LC-MS/MS proteomic characterization of biomarkers associated with inflammation in two studies in patients with advanced colorectal cancer (stage III and IV), followed by validation in a second colorectal cancer study, revealed complement C5 as a suitable biomarker for diagnosis; *SAA4* mRNA was only present in colorectal cancer but not in normal tissue, while SAA4 protein could act as a subtle marker of tumorigenesis [103].

An excellent overview of nanosized membrane-enclosed sacs (i.e., small extracellular vesicles) has covered an optional quantitative proteomic perspective for clinical cancer research [104]. Proteins associated with small extracellular vesicles were found to be associated with inflammation, lipoprotein metabolism, and cell adhesion, and some of these proteins, such as von Willebrand factor, apo A-IV, inter-alpha-trypsin inhibitor heavy chain H4, antithrombin (coded by *serpinc1*) and SAA4, respectively, have been identified as potential biomarkers in a panel of different types of cancer [104].

### 8.2. SAA4: A Potent Bio- or Surrogate-Marker?

To identify treatment strategies for idiopathic pulmonary arterial hypertension (a rare disease with poor prognosis and a limited responsiveness to therapy), a plasma proteomic approach revealed serum amyloid P and SAA4 as potential clinically relevant parameters to differentiate between children with a poor or good outcome to therapy [105].

Another approach to identify novel cerebrospinal fluid biomarkers in neurodegeneration, Heywood and colleagues revealed (out of 19 proteins significantly increased in both Lewy Body dementia and Alzheimer’s disease—compared to controls) four potential biomarkers, such as GM2-activator protein, malate dehydrogenase, prosaposin, and SAA4, respectively [106]. Within a standard clinical assessment and neuroimaging overlook in 45 patients with Alzheimer’s disease (compared with 45 age- and sex-matched controls), 48 target peptides out of 14 proteins identified by parallel-reaction monitoring revealed a panel of eight proteins (apo A-IV, carboxypeptidase B2, coagulation factor X, complement CIs, immunoglobulin heavy constant mu, platelet factor 4, pro-platelet basic protein, and SAA4) and demographic characteristic for these markers with an AUC of 0.923 [107].

A previous report addressed the question of whether SAA4 might act as an indicator of hepatic protein synthesis and/or even as an indicator of nutrition [108]. Plasma proteomic analyses from post-surgical gastrointestinal cancer patients receiving either standard or immunomodulatory enteral nutrition identified different key pathways favoring different outcome for patients; while the latter diet group revealed lower complications and improved clinical aspects (paralleled by a broad spectrum of proteins related to innate/adaptive immunity such as complement proteins C3, C5, and C9, proteins involved in cell repair, lipid metabolism and others), the further diet group revealed higher complications leading to pro-inflammatory and hypercoagulable states like sepsis and thrombosis paralleled by increased levels of acute-phase reactants haptoglobin, fibrinogen and SAA4) [109].

Rhegmatogenous retinal detachment associated with choroidal detachment, a subtype of Rhegmatogenous retinal detachment, is characterized by uveal inflammation, low intraocular pressure, and disruption of the blood–brain barrier. Following proteomic analyses, out of 237 differentially expressed proteins, 63 were found to be upregulated; following ELISA validation, the top five upregulated proteins and/or potential biomarkers were apo C-II, inter-alpha-trypsin heavy chain H1, inter-alpha-trypsin heavy chain H2, vitronectin, and SAA4 [110]. Most importantly, SAA4 may also be considered a fluctuating marker in Rhegmatogenous retinal detachment associated with choroidal detachment, as it shows dynamic changes in the inflammatory response at different stages, when comparing months one and six [110]. A parallel study concentrated on the efficacy of hormonal intervention treatment of retinal detachment. Out of 295 differentially expressed proteins (classified within a panel of regulatory pathways) in the vitreous humor of Rhegmatogenous retinal detachment associated with choroidal detachment collectives, vitronectin and apo D were found to positively correlate with postoperative best-corrected visual acuity, while apo D, ubiquitin-conjugated enzyme E2 variant and SAA4 may act as potential biomarkers for this disease [111]. Using the LC-MS/MS technique to reveal common marker(s) for choroidal vasculopathies, SAA4 was present among the ten most upregulated proteins present in the aqueous humor in both age-related macular degeneration (n = 10) and polypoidal vasculopathy (n = 10) compared to controls (subjects with cataracts n = 10). Both validations of SAA4 revealed significantly higher levels in both vasculopathy groups, as supported by respective ROC curves for SAA4 reaching AUC levels of 0.92 and 0.95 [112].

A comprehensive analysis of *Plasmodium falciparum*-mediated changes in the human proteome (severe malaria patients (n = 37); non-severe malaria patients (n = 67); healthy controls (n = 146)) performed in three different endemic regions in India revealed a 1.4-fold change (controls vs. non-severe) and 1.8-fold change (controls vs. severe) for SAA4 protein (Uniprot accession number 35542) [113]. Another study attributed to the spectrum of alternative medicine (concentrating on malignant tumors classified according to Uighur medicine) revealed SAA4 to rank at position eight on a list of differentially expressed proteins when comparing the ratio of abnormal Savda type tumor vs. non-abnormal Savda type tumor (including other syndromes) [114].

A quite comprehensive approach to characterize disease biomarkers in acute graft-versus-host disease (grade II) revealed four differentially expressed protein spots; while coagulation factor XIII β chain and plasminogen were found less abundant in patients’ plasma, fibrinogen β chain fragment and SAA4 (in particular on two different time intervals) were found to be predominantly expressed [115]. Using a multiplex protein assay based on UHPLC-selected reaction monitoring to quantify acute-phase proteins from dry blot spots has been successfully established for α-1-acid glycoprotein, α-1 antitrypsin, C-reactive protein, A-SAA, and SAA4, respectively [116].

Rheumatoid arthritis, a chronic autoimmune disease (characterized by synovitis, influx of monocytes and polymorphonucleocytes into synovial fluid), is commonly known to promote inflammation-mediated alterations in physicochemical and metabolic properties of circulating lipoproteins. Both A-SAA and C-SAA were present in synovial fluids, and levels of SAA4 in synovial fluids ranged between 17 and 214 μg/mL as measured by ELISA [117]; mean serum SAA4 concentrations in arthritis patients were 106 ± 49 μg/mL, findings in good accordance with previous reports [34,36]. Inhibition of IL-6Rα by monoclonal antibody tocilizumab for 12 weeks resulted in a decrease in C-reactive protein (approx. 80%) as well as complement C4 protein and SAA4 (approx. 30%), besides changes in other markers [118]. In a pre-screening approach by LC-MS/MS, SAA4 was significantly upregulated in rheumatoid arthritis (compared to controls) with a higher area under the curve than C-reactive protein and also positively correlated with levels of rheumatoid factor [119]. A parallel study using LC-MS/MS analyses and ELISA validation revealed five proteins (out of 38 selected) that were statistically differentially expressed between rheumatoid arthritis patients and controls: gelsolin, plasminogen, retinol binding protein, and vitamin binding protein were downregulated, while SAA4 was upregulated [120]. Analysis of serum proteins from rheumatoid arthritis patients, starting with 435 identified proteins, revealed five highly expressed candidates (angiotensinogen, complement C3, the protease inhibitor kallistatin, vitamin D-binding protein, and SAA4, respectively) compared to controls. Further verification by multiple reaction monitoring method revealed the latter two proteins with an AUC > 0.8 (0.85 and 0.83) and a classification accuracy of 81.4% and 86.0% in patients and healthy controls [121]. An ongoing study from the same group focused on an extended and more specific panel of serum biomarkers for diagnosis of rheumatoid arthritis by addressing process network analysis for differently expressed proteins between patients (n = 251) and controls (n = 230) leading to seven candidate biomarkers; only four proteins (angiotensinogen, retinol binding protein, vitamin binding protein and SAA4) revealed AUC levels > 0.8 and most importantly showed a high classification accuracy irrespective of the patients status, i.e., rheumatoid factor-negative or -positive [122]. An excellent cross-sectional study including two different cohorts (n = 25 each) of early (<2 years) versus established (>20 years) rheumatoid arthritis revealed a bulk of 20 proteins commonly expressed; out of them, only six candidate biomarkers (C-C motif chemokine 18, Cathepsin L1, sortilin, beta-galactoside alpha-2,6-sialyltransferase 1, tumor necrosis factor receptor superfamily member 10A, and SAA4) could effectively differentiate between patients and healthy controls. Following a six-month treatment of rheumatoid arthritis patients to get an idea of changes within the cardiometabolic and cardiovascular proteome, a six-month treatment of patients with methotrexate (<2 years) or tofacitinib (>20 years) revealed only two proteins, C-C motif chemokine 18 and SAA4, as a proper response to therapy [123]. Whether hard data will finally be published on the optional capacity of SAA4 to act as a definitive diagnostic marker for rheumatoid arthritis, combined with anti-cyclic citrullinated protein antibody, remains to be seen [124].

Within a pharmaco-proteomic approach (performed in 128 prepubertal children), the basic question was to reveal optional protein biomarkers related to either anabolic and/or lipolytic processes in relation to changes in different body compartments (such as bone mass or longitudinal growth) after a one-year treatment period with growth hormones. Analyses were performed by SELDI-TOF-MS and dual-energy X-ray absorptiometry. Among proteins, representing real nutrition markers, transthyretin (previously called pre-albumin), apo C-I, apo A-II, and SAA4, respectively, ranked first [125,126,127].

In another clinical study comparing the serum proteome from 35 amnestic patients (suffering from mild cognitive impairment) and 35 cognitively healthy controls, five proteins (complement factor B, carboxypeptidase N subunit 2, galectin-3 binding protein, serum amyloid P-component, and SAA4, respectively) were significantly decreased in the patient group; only the latter one had a direct link to dementia while the role of SAA4 still seems to remain an enigma in this disease [128]. Following serum protein alterations in patients with major depressive disorders, the combination of four proteins, namely antithrombin-III, complement C1q subcomponent subunit B, serum amyloid P-component and SAA4, was considered a unique biomarker collective; it apparently can differentiate between (i) depression and remission states of patients as well as (ii) remission states of residual impairment and complete remission [129]. Whether expression levels of blood plasma markers (apo C-III, apo D, SAMP, Saa1, and Saa4) found in depressed mice under reported chronic social stress conditions may represent any predisposition to psychoemotional disorders in humans remains an open question [130].

Using LC-ESI-QToF-MS/MS analysis, five inflammatory/regulatory as well as vascular integrity proteins were found to be deregulated in two migraine groups (MM group: patients with menstrual-related migraine without taking hormonal contraceptives; PM group: patients having suffered from menstrual-related migraine before menopause) compared to a headache group; while prothrombin, serum amyloid P and Igκ chain C region were upregulated, apo A-I and SAA4 were downregulated [131].

Combining a panel of longitudinal proteomic datasets from three different clinical trials (S3WP-T2D, Ramp and IMPOCT) and confirmation of statistically significant findings in two additional cohorts (IMI-DIRECT and IMI-RHAPSODY) with type II diabetes (either untreated or treated with metformin) revealed that out of 23 proteins, eleven were associated with metformin exposure, while 12 other proteins (including SAA4) were replicated using the discovery Olink platform [132].

Another study included 30 patients to reveal proper proteins related to coronary heart disease (including acute myocardial infarction and stable coronary artery disease); out of 907 quantifiable proteins, albumin and sex hormone-binding globulin, as well as four apolipoproteins (apo C-II, apo C-III, apo C-IV and SAA4) related to lipid metabolism by modulating the functional capacity of HDL particles were found [133]. Lipid and lipoprotein metabolism undergo major changes in patients suffering from sepsis. Sharma and colleagues [134] focused on the proteome in these patients secondary to acquired pneumonia during their stay in the hospital. Besides acute inflammation and changes in homeostasis and blood coagulation, impaired levels of proteins primarily associated with the HDL particle, such as paraoxonase 1 and four apolipoproteins, including apo L-I, apo C-I, apo A-IV, and SAA4, were found; validation in hospital-acquired patients revealed a decrease in lipoprotein-associated cholesterol as well as major apolipoproteins present in HDL and LDL particles [134]. Sarkar and colleagues published an excellent overview of biomarkers in type I diabetes that may affect circulating proteins involved in immune response, complement activation, and lipid metabolism. Following strict inclusion criteria, identification of 266 unique proteins with 11.6% identified across three or more studies out of 13 revealed a major subset of 17 proteins, including SAA4 [135]. Non-alcoholic fatty acid liver disease patients with advanced fibrosis (n = 26, stage F3-F4) have a high prevalence for type II diabetes, a high cardiovascular coronary artery disease index, a low number of HDL particles and in parallel decreased number of five HDL-associated proteins including lecithin-cholesterol acyl-transferase and four apolipoproteins (apo C-I, apo C-IV, apo M, and SAA4, respectively) when compared to non-alcoholic fatty acid liver disease patients (n = 114; stage F0-F2) [136]. Although four adult hypertrophic cardiomyopathy markers (talin 1, complement C3a, thrombospondin and aldolase fructose-biphosphate) and three more recent markers (profilin 1, lipoprotein(a) and glycogen phosphorylase B) have been implemented for disease diagnosis; this seven-biomarker diagnostic panel for childhood onset hypertrophic cardiomyopathy has been upgraded by a panel of four additional proteins (immunoglobulin heavy constant epsilon (*p* < 0.001), complement 5B (*p* < 0.001), apo L1 (*p* < 0.041) and SAA4) (*p* < 0.015)) reported to be significantly impaired in a cohort of children suffering from high sudden cardiac death risk when compared to children with either low or intermediate sudden cardiac death risk [137]. In a well-designed clinical case–control study in pediatric patients (n = 33) with hemophagocytic lymphohistiocytosis and three disease control groups (sepsis, n = 43; pediatric intensive care group non infected, n = 39; Ebstein-Barr virus, n = 21) proteomic comparison within all groups revealed that hemophagocytic lymphohistiocytosis is characterized by subtle changes in several pathways and cascades leading to eight differentially expressed proteins including albumin, apo A-I, extracellular matrix protein 1, fibrinogen beta chain, fibrinogen gamma chain, plastin-2, vascular cell adhesion protein 1, and SAA4, respectively [138].

## 9. SAA4: General Findings Related to SAA4′s Function

Following incubation of serum with adult parasitic worms (*Schistosoma mansoni* for only one hour) to reveal their impact on the human proteome, 2D-DIGE and immunoblot analyses revealed eleven (out of 20) proteins to be associated with the complement system, while the remaining fraction, among others, contained human α2-macroglobulin, actin cytoplasmic 2, and SAA4, respectively [139].

Although increased serum levels of SAA2 and SAA4 have been measured in patients with Guillain-Barré syndrome (including demyelination (n = 21) and axonal subtypes (n = 19)), values more likely seem to represent acute-inflammatory stages but not necessarily potential biomarkers to mirror the severity of myelination injury [140].

Observations that SAA4 plasma levels (including (non)glycosylated SAA4 forms, 14 and 19 kDa) were higher in venous thrombosis patients compared to controls (48.1 μg/mL versus 38.4 μg/mL) and that recombinant SAA4 (i) enhances clotting in plasma and (ii) increases activation of prothrombin suggest a likely role of SAA4 to play within procoagulant activity [141].

Although only reported as a single case study: dried blood spot proteomic analysis (samples taken before, during and after the race) from a 40-year-old athlete, participating in the “Race Across America” (10.1 days, 4941 km) revealed the largest increase in protein complement component 7, complement C4-B, and SAA4 in the following order: 359% > 231% > 210% [142].

Besides expression of vascular adhesion molecule-1 and production of nitric oxide, HDL acts as an eminent player during reverse cholesterol transport from peripheral tissues after esterification back to the liver; six proteins (alpha-1B-glycoprotein, angiotensinogen, plasma serine protease inhibitor, thyroxine-binding globulin, vitronectin and SAA4) were found to be more abundantly present (ratio > 2-fold) in HDL particles with the highest capacity to promote cellular cholesterol efflux [143]. Whether this capacity will be mediated via direct interaction of SAA4 with scavenger receptor class B, type I or even in an ATP-binding cassette transporter-(in)dependent manner, as reported for A-SAA proteins and/or their extent of lipidation, remains to be further investigated [144,145].

Whether observations that clusterin and SAA4 may serve as biomarker targets for chronic alcohol intake in humans remains to be further investigated [146].

## 10. Optional Studies to Clarify the Function of SAA4 in Future Studies

As A-SAA is still considered the prime acute-phase reactant within the SAA (super)family, a valid question arises on potential functions of SAA4 besides its more or less involvement in inflammation or its contribution to cancer metabolism as a potent or even independent disease marker.

A promising approach undoubtedly includes the identification of so-called senescence-associated genes that, in context with inflammatory cytokines and growth factors, may promote malignant invasion, proliferation, and progression of various types of cancer. Coppola and colleagues [147] concentrated on a senescence-associated gene signature with a major focus on progression and prognosis in human glioma. Out of 14 senescent genes (identified in 47 patients with glioma of older age), the highest senescence score in gliosarcoma revealed six genes (correlating with age) and two genes (correlating with prognosis), both including *SAA4* and addressing its role in metastasis, inflammation, and angiogenesis. In line with data mentioned above, qRT-PCR and chemokine protein arrays addressed *SAA4* (besides *E-cadherin*, *fibrinogen*, *IL-8*, and *IL-32*) as a major player involved in the regenerative capacity of hepatocytes during chronic liver diseases, where so-called “growth-arrested” cells may shift into a senescent stage in an in vitro model of cells to generate a liver-derived senescence-associated secretory phenotype [148]. Although a huge prospective observational cohort study of musculoskeletal health in 5994 men (≥65 years), focusing on peptides/proteins, including SAA4, being associated with mortality in discovery and replication phases; this finding might definitively support a candidate role of SAA4 during senescence, a topic that is worth further investigation [149].

A major task to reveal physiological function of human SAA4 and to compare proper activities of (non)glycosylated SAA4 proteins includes a well-designed expression system for both proteins, detailed knowledge on exact glycan residues and finally a suitable strategy to reach an optional cleavage efficiency (depending on the respective tag) to generate non-glycosylated SAA4 (14 kDa); first attempts to identify glycan structures on HDL particles have already been reported [45]. Expression of SAA4 in *Escherichia coli* has been previously published, but in contrast to SAA1, SAA4 did not induce lysis of bacterial cells [150]. Although expression of His_6_-tagged SAA4 protein (confirmed by immunoblot analysis using a panel of sequence-specific SAA4 antibodies as well as anti-His_6_ epitope antibodies) after a silent mutation in secondary rbs-like sequences did work, it resulted in moderate cleavage efficiency of the tagged SAA4 [151]. As expression in *Escherichia coli* does not glycosylate proteins, some of us previously succeeded in expressing the SAA4 protein in yeast (Herz, Windpassinger and Malle, unpublished data); however, *Pichia pastoris* is known to glycosylate in a different manner than the human system [152], so humanized glycosylation in *Pichia pastoris* might represent a favorable approach to solve these problems [152,153,154].

Whether the expression of SAA4 in hepatoma cell lines (including cell lines other than HepG2) may be considered a suitable approach for the generation and isolation of SAA4, the question still remains whether expression levels will merit this procedure [51]. An important but major question still remains whether bacterial contamination, as reported for recombinant SAA proteins (mimicking potential interactions in vitro via candidate surface receptors, Toll-like receptor 2/4), will affect the biological activities of the expressed SAA4 protein [155,156].

An elegant approach that deserves further studies refers to differences between *Saa^+/+^* and *Saa^−/−^* mice regarding (un)balanced inflammation in both strains in response to sterile inflammation, as well as different expression of Saa1/2, Saa3, and Saa4 in wild-type mice, addressing subtle differences in macrophages expressing all four murine Saa proteins in response to either bacterial- (*Pseudomonas aeruginosa*) or lipopolysaccharide-mediated infection [157].

Rats also express *A-SAA*-related genes with striking homology to human/murine *SAA1*/*SAA2*; however, the respective sequence coding for an approximately 50 amino acid region in the N-terminal portion of the predicted A-SAA protein is lacking [158]. As rats neither express HDL-associated A-SAA proteins nor have been found to suffer from reactive systemic amyloid A amyloidosis (characterized by deposition of the 76 amino acid N-terminal degradation product from A-SAA in various organs after cleavage) [159], so far, SAA4 is the only functional Saa4 protein in rats [160]. Most importantly, transcription factors (in particular members of the C/EBP family) responsible for SAA expression in general [161] may be of outstanding importance to mediate expression of rat SAA4, as C/EBPα knockout in mice led to the complete absence of *Saa4* mRNA [160,162]. So, the question comes up: What is the function of rat Saa4 under physiological conditions? Will it compensate for the lack of A-Saa proteins normally generated during the acute-phase response, or will it solely mimic or underscore the role of SAA4 observed in human diseases, including malignancies? Both knocking out rat *Saa4* and expressing intact either human or murine *SAA1/2* genes might be considered an exciting approach.

Amyloidosis has also been found to be associated with a mutated SAA4 protein [163], although recombinant SAA4 has originally been reported not to show positive Congo red staining and fibrillar structures [164]. This observation might be worth investigating further, as two different mutations (W22G and C71Y) in human SAA4 have been previously reported in patient amyloid deposits (addressing pathogenic mutations of hereditary amyloidosis) [165].

To obtain a more detailed and proper information on the optional function of SAA4, which is not necessarily conclusive to be obtained from rodents; pigs sharing striking homology with humans, not only on the genetic level but also regarding biological similarities (including organ size and function and optional xenotransplantation), might be considered a more meaningful animal system to reveal SAA4′s function. Although remarkable differences exist between the human and the pig SAA gene cluster, hepatic transcription of SAA4 in pigs is minimally increased in response to bacterial infection with *Staphylococcus aureus*, but markedly upregulated in the presence of *Actinobacillus pleuropneumoniae* [166]. In a well-designed mechanistic study, Sallustio and colleagues [167] focused on endothelial dysfunction, considered a hallmark of sterile-induced acute kidney injury [167]. Authors convincingly demonstrated that human renal progenitor cells were found to effectively revert lipopolysaccharide-induced endothelial to mesenchymal transition, three proteins (granulocyte C-X-C motif chemokine ligand 6, BPI fold-containing family A member 2, and SAA4) played a major role during this process; data that finally support the notion that SAA4 may be considered an inducible SAA protein in response to lipopolysaccharide and may further be considered an antiseptic protein to counteract lipopolysaccharide-mediated effects [167].

Julian and colleagues [168] were the first to use discovery-based proteomic methods to identify optional biomarkers to compare acute mountain sickness-susceptible and -resistant subjects after exposure to hypobaric hypoxia (9 h, 4875 m, barometric pressure, 425 mm Hg). Serum amyloid P component, insulin-like growth factor-binding protein complex acid labile chain, complement component 7, mannose binding protein C, von Willebrand factor, apo C-I and SAA4 (even listed twice within two gene ontology categories) were identified in the latter group [168]; a study that apparently paved the way for archaic ancestry estimation of genes (including *SAA4*) in the highlander (approx. 1200 to 4500 m above sea level) population for mountain sickness in Papua New Guinea [169] and maybe might also be adapted to other mountainous areas worldwide or even during space shuttle flights, to get further information on extending the role of SAA4.

An interesting approach, i.e., intraplantar injection of snake venom from *Bothrops erythromelas* (5.0 µg) in mice, showed a maximum increase in paw volume after 1 h and parallel increases in epidermal thickness. Analysis of plasma samples (taken 30 min after injection) and subsequent proteome alterations by LC-MS/MS revealed six potential biomarkers: three (factor XII, fibulin, and vitamin K-dependent protein Z) downregulated and three others (adiponectin, apo A-I, and SAA4) upregulated. This approach represents not only short-time plasma protein alterations of apo A-I (commonly but not always considered a negative acute-phase reactant [170]) and SAA4 (apparently acting as a positive acute-phase reactant) but might also refer to snake-bite-dependent protein-generated dynamics as an example within a series of other poisonous venoms that, when antagonized in time, will save human life [171].

## 11. Concluding Remarks

The primary objective of this overview was to summarize data on human SAA4 being published in a panel of clinical studies related to (non)inflammatory conditions and/or different malignancies. Indeed, SAA4 might be considered a young sprout within the SAA family in the mammalian system, and it will be a long and winding road to achieve the same scientific credit as obtained over decades for SAA1/2, considered prime acute-phase reactants in the mammalian inflammatory system. At least a further series of clinical data, as well as molecular biology experiments and hard biochemical data, may represent the basis for further potential functions of mammalian SAA4 proteins. At least the fact that SAA4 has been confirmed as a potent clinical (bio)marker in cancer research may be considered promising [104].

To conclude, the findings reviewed above will no longer justify that SAA4 may be considered “C-SAA” or “constitutively expressed SAA protein” and do support the notion that SAA4 may be considered an “underdog within the whole SAA family”. While this seems to represent the good side of the coin, we, however, are aware of the current state of evidence as well as official data from the OMIM database (https://www.omim.org, accessed on 16 March 2026), indicating that there is no convincing evidence associating SAA4 with any inherited disease in humans.

## Figures and Tables

**Table 1:** Percent identity matrix of SAA4 proteins—created by Clustal2.1—between different mammalian species listed in Figure 2.

	*Rhino*	*Homo*	*Gorilla*	*Pan_tro*	*Pan_pan*	*Mus*	*Rattus*
*Rhinopithecus*	100.00	84.62	85.38	85.38	84.62	56.92	54.62
*Homo*	84.62	100.00	98.46	98.46	97.69	57.69	56.15
*Gorilla*	85.38	98.46	100.00	99.23	98.46	57.69	56.15
*Pan_troglodytes*	85.38	98.46	99.23	100.00	99.23	57.69	56.15
*Pan_paniscus*	84.62	97.69	98.46	99.23	100.00	57.69	56.15
*Mus*	56.92	57.69	57.69	57.69	57.69	100.00	83.08
*Rattus*	54.62	56.15	56.15	56.15	56.15	83.08	100.00

**Table 2:** Overview of allele counts, numbers, and frequency of rs2460827 in different populations or ethnic groups.

Genetic Ancestry Group	Allele Count	Allele Number	Number of Homozygotes	Allele Frequency
Amish	912	912	456	1.000
Europe (Finnish)	63,161	64,040	31,148	0.9863
Europe (non-Finnish)	1,149,941	1,180,022	560,342	0.9745
Admixed American	57,952	59,996	27,998	0.9659
South Asian	87,811	91,070	42,365	0.9642
Ashkenazi Jewish	28,333	29,604	13,554	0.9571
Remaining	59,212	62,500	28,115	0.9474
Middle Eastern	5629	6062	2620	0.9286
East Asian	40,376	44,866	18,165	0.8999
African/African American	55,262	74,904	20,438	0.7378
XX	778,706	812,346	374,442	0.9586
XY	769,883	801,630	370,759	0.9604
**Total**	**1,548,589**	**1,613,976**	**745,201**	**0.9595**

## Data Availability

No new data were created or analyzed in this study.

## References

[1] Sack G.H. (2018). Serum amyloid A—A review. Mol. Med..

[2] Uhlar C.M., Whitehead A.S. (1999). Serum amyloid A, the major vertebrate acute-phase reactant. Eur. J. Biochem..

[3] Kluve-Beckerman B., Dwulet F.E., Benson M.D. (1988). Human serum amyloid A. Three hepatic mRNAs and the corresponding proteins in one person. J. Clin. Investig..

[4] Upragarin N., Landman W.J., Gaastra W., Gruys E. (2005). Extrahepatic production of acute phase serum amyloid A. Histol. Histopathol..

[5] Malle E., Steinmetz A., Raynes J.G. (1993). Serum amyloid A (SAA): An acute phase protein and apolipoprotein. Atherosclerosis.

[6] Malle E., De Beer F.C. (1996). Human serum amyloid A (SAA) protein: A prominent acute-phase reactant for clinical practice. Eur. J. Clin. Investig..

[7] Moccia V., Tucciarone C.M., Garutti S., Milazzo M., Ferri F., Palizzotto C., Mazza M., Basset M., Zini E., Ricagno S. (2025). AA amyloidosis in vertebrates: Epidemiology, pathology and molecular aspects. Amyloid.

[8] Zhou J., Dai Y., Lin Y., Chen K. (2022). Association between serum amyloid A and rheumatoid arthritis: A systematic review and meta-analysis. Semin. Arthritis Rheum..

[9] Getz G.S., Krishack P.A., Reardon C.A. (2016). Serum amyloid A and atherosclerosis. Curr. Opin. Lipidol..

[10] Malle E., Sodin-Semrl S., Kovacevic A. (2009). Serum amyloid A: An acute-phase protein involved in tumour pathogenesis. Cell Mol. Life Sci..

[11] den Hartigh L.J., May K.S., Zhang X.S., Chait A., Blaser M.J. (2023). Serum amyloid A and metabolic disease: Evidence for a critical role in chronic inflammatory conditions. Front. Cardiovasc. Med..

[12] Kluve-Beckerman B., Drumm M.L., Benson M.D. (1991). Nonexpression of the human serum amyloid A three (SAA3) gene. DNA Cell Biol..

[13] Chait A., Wang S., Goodspeed L., Gomes D., Turk K.E., Wietecha T., Tang J., Storey C., O’Brien K.D., Rubinow K.B. (2021). Sexually Dimorphic Relationships Among Saa3 (Serum Amyloid A3), Inflammation, and Cholesterol Metabolism Modulate Atherosclerosis in Mice. Arter. Thromb. Vasc. Biol..

[14] Sanada Y., Yamamoto T., Satake R., Yamashita A., Kanai S., Kato N., van de Loo F.A., Nishimura F., Scherer P.E., Yanaka N. (2016). Serum Amyloid A3 Gene Expression in Adipocytes is an Indicator of the Interaction with Macrophages. Sci. Rep..

[15] den Hartigh L.J., Wang S., Goodspeed L., Ding Y., Averill M., Subramanian S., Wietecha T., O’Brien K.D., Chait A. (2014). Deletion of serum amyloid A3 improves high fat high sucrose diet-induced adipose tissue inflammation and hyperlipidemia in female mice. PLoS ONE.

[16] Yang X.Y., Liu Y., Li W., Li H.X., Zhong Y.W., Cao Y.H., Liu T.Y., Xu A.J., Gu W., Liang Q. (2025). Serum amyloid A3 aggravates bleomycin-induced pulmonary fibrosis through Kruppel-like factor 6-dependent interlukin-36alpha expression. Acta Pharmacol. Sin..

[17] Whitehead A.S., de Beer M.C., Steel D.M., Rits M., Lelias J.M., Lane W.S., de Beer F.C. (1992). Identification of novel members of the serum amyloid A protein superfamily as constitutive apolipoproteins of high density lipoprotein. J. Biol. Chem..

[18] Watson G., Coade S., Woo P. (1992). Analysis of the genomic and derived protein structure of a novel human serum amyloid A gene, SAA4. Scand. J. Immunol..

[19] Steel D.M., Sellar G.C., Uhlar C.M., Simon S., DeBeer F.C., Whitehead A.S. (1993). A constitutively expressed serum amyloid A protein gene (SAA4) is closely linked to, and shares structural similarities with, an acute-phase serum amyloid A protein gene (SAA2). Genomics.

[20] Sellar G.C., Whitehead A.S. (1993). Localization of four human serum amyloid A (SAA) protein superfamily genes to chromosome 11p: Characterization of a fifth SAA-related gene sequence. Genomics.

[21] Uhlar C.M., Burgess C.J., Sharp P.M., Whitehead A.S. (1994). Evolution of the serum amyloid A (SAA) protein superfamily. Genomics.

[22] de Beer M.C., de Beer F.C., Gerardot C.J., Cecil D.R., Webb N.R., Goodson M.L., Kindy M.S. (1996). Structure of the mouse Saa4 gene and its linkage to the serum amyloid A gene family. Genomics.

[23] Zámocký M., Ferianc P. (2023). Discovering the deep evolutionary roots of serum amyloid A protein family. Int. J. Biol. Macromol..

[24] Madeira F., Madhusoodanan N., Lee J., Eusebi A., Niewielska A., Tivey A.R.N., Lopez R., Butcher S. (2024). The EMBL-EBI Job Dispatcher sequence analysis tools framework in 2024. Nucleic Acids Res..

[25] Marhaug G., Husby G., Dowton S.B. (1990). Mink serum amyloid A protein. Expression and primary structure based on cDNA sequences. J. Biol. Chem..

[26] Sellar G.C., DeBeer M.C., Lelias J.M., Snyder P.W., Glickman L.T., Felsburg P.J., Whitehead A.S. (1991). Dog serum amyloid A protein. Identification of multiple isoforms defined by cDNA and protein analyses. J. Biol. Chem..

[27] Syversen P.V., Sletten K., Marhaug G., Husby G., Lium B. (1993). The Revised Amino Acid Sequence of Serum Amyloid A (SAA) Protein in Mink. Scand. J. Immunol..

[28] Badolato R., Johnston J.A., Wang J.M., McVicar D., Xu L.L., Oppenheim J.J., Kelvin D.J. (1995). Serum amyloid A induces calcium mobilization and chemotaxis of human monocytes by activating a pertussis toxin-sensitive signaling pathway. J. Immunol..

[29] Pruzanski W., de Beer F.C., de Beer M.C., Stefanski E., Vadas P. (1995). Serum amyloid A protein enhances the activity of secretory non-pancreatic phospholipase A2. Biochem. J..

[30] Malle E., Bollmann A., Steinmetz A., Gemsa D., Leis H.J., Sattler W. (1997). Serum amyloid A (SAA) protein enhances formation of cyclooxygenase metabolites of activated human monocytes. FEBS Lett..

[31] Raynes J.G., McAdam K.P.W.J. (1991). Serum Amyloid A Isoforms in Inflammation. Scand. J. Immunol..

[32] Ducret A., Bruun C.F., Bures E.J., Marhaug G., Husby G., Aebersold R. (1996). Characterization of human serum amyloid A protein isoforms separated by two-dimensional electrophoresis by liquid chromatography/electrospray ionization tandem mass spectrometry. Electrophoresis.

[33] Farwig Z.N., McNeal C.J., Little D., Baisden C.E., Macfarlane R.D. (2005). Novel truncated isoforms of constitutive serum amyloid A detected by MALDI mass spectrometry. Biochem. Biophys. Res. Commun..

[34] Yamada T., Kluve-beckerman B., Kuster W.M., Liepnieks J.J., Benson M.D. (1994). Measurement of serum amyloid A4 (SAA4): Its constitutive presence in serum. Amyloid.

[35] Yamada T., Wada A., Yamaguchi T., Itoh Y., Kawai T. (1997). Automated measurement of a constitutive isotype of serum amyloid A/SAA4 and comparison with other apolipoproteins. J. Clin. Lab. Anal..

[36] de Beer M.C., Yuan T., Kindy M.S., Asztalos B.F., Roheim P.S., de Beer F.C. (1995). Characterization of constitutive human serum amyloid A protein (SAA4) as an apolipoprotein. J. Lipid Res..

[37] Sun H.-Y., Chen S.-F., Lai M.-D., Chang T.-T., Chen T.-L., Li P.-Y., Shieh D.-B., Young K.-C. (2010). Comparative proteomic profiling of plasma very-low-density and low-density lipoproteins. Clin. Chim. Acta.

[38] Bancells C., Canals F., Benítez S., Colomé N., Julve J., Ordóñez-Llanos J., Sánchez-Quesada J.L. (2010). Proteomic analysis of electronegative low-density lipoprotein. J. Lipid Res..

[39] Hoofnagle A.N., Heinecke J.W. (2009). Lipoproteomics: Using mass spectrometry-based proteomics to explore the assembly, structure, and function of lipoproteins. J. Lipid Res..

[40] Vaisar T., Pennathur S., Green P.S., Gharib S.A., Hoofnagle A.N., Cheung M.C., Byun J., Vuletic S., Kassim S., Singh P. (2007). Shotgun proteomics implicates protease inhibition and complement activation in the antiinflammatory properties of HDL. J. Clin. Investig..

[41] Davidson W.S., Silva R.A.G.D., Chantepie S., Lagor W.R., Chapman M.J., Kontush A. (2009). Proteomic Analysis of Defined HDL Subpopulations Reveals Particle-Specific Protein Clusters. Arterioscler. Thromb. Vasc. Biol..

[42] Yamada T., Miida T., Itoh Y., Kawai T., Benson M.D. (1996). Characterization of serum amyloid A4 as a plasma apolipoprotein. Clin. Chim. Acta.

[43] Kindy M.S., de Beer M.C., Yu J., de Beer F.C. (2000). Expression of mouse acute-phase (SAA1.1) and constitutive (SAA4) serum amyloid A isotypes: Influence on lipoprotein profiles. Arter. Thromb. Vasc. Biol..

[44] Heinecke J.W. (2009). The HDL proteome: A marker--and perhaps mediator--of coronary artery disease. J. Lipid Res..

[45] Krishnan S., Huang J., Lee H., Guerrero A., Berglund L., Anuurad E., Lebrilla C.B., Zivkovic A.M. (2015). Combined High-Density Lipoprotein Proteomic and Glycomic Profiles in Patients at Risk for Coronary Artery Disease. J. Proteome Res..

[46] Yamada T., Kakihara T., Kamishima T., Fukuda T., Kawai T. (1996). Both acute phase and constitutive serum amyloid A are present in atherosclerotic lesions. Pathol. Int..

[47] Radovani B., Gudelj I. (2022). N-Glycosylation and Inflammation; the Not-So-Sweet Relation. Front. Immunol..

[48] Pasala C., Sharma S., Roychowdhury T., Moroni E., Colombo G., Chiosis G. (2024). N-Glycosylation as a Modulator of Protein Conformation and Assembly in Disease. Biomolecules.

[49] Esmail S., Manolson M.F. (2021). Advances in understanding N-glycosylation structure, function, and regulation in health and disease. Eur. J. Cell Biol..

[50] Karlsson H., Leanderson P., Tagesson C., Lindahl M. (2005). Lipoproteomics II: Mapping of proteins in high-density lipoprotein using two-dimensional gel electrophoresis and mass spectrometry. Proteomics.

[51] Takarada T., Fujinaka R., Shimada M., Fukuda M., Yamada T., Tanaka M. (2025). Effect of N-glycosylation on secretion, degradation and lipoprotein distribution of human serum amyloid A4. Biochim. Biophys. Acta Mol. Cell Biol. Lipids.

[52] Savinova O.V., Fillaus K., Jing L., Harris W.S., Shearer G.C. (2014). Reduced Apolipoprotein Glycosylation in Patients with the Metabolic Syndrome. PLoS ONE.

[53] Yamada T., Sato J., Kotani K., Tanaka M. (2014). Influence of polymorphism on glycosylation of serum amyloid a4 protein. Biochem. Res. Int..

[54] Jönsson M., Bäckryd E., Ljunggren S., Ottosson N., Jauregi-Miguel A., Liin S.I., Checa A., Wheelock C.E., Ghafouri B. (2025). System-wide targeted analysis of oxylipins and lipoproteins in chronic peripheral neuropathic pain-an explorative study. Pain. Rep..

[55] Alfakry H., Malle E., Koyani C.N., Pussinen P.J., Sorsa T. (2016). Neutrophil proteolytic activation cascades: A possible mechanistic link between chronic periodontitis and coronary heart disease. Innate Immun..

[56] Ljunggren S., Bengtsson T., Karlsson H., Starkhammar Johansson C., Palm E., Nayeri F., Ghafouri B., Davies J., Svensäter G., Lönn J. (2019). Modified lipoproteins in periodontitis: A link to cardiovascular disease?. Biosci. Rep..

[57] Lönn J., Ljunggren S., Klarström-Engström K., Demirel I., Bengtsson T., Karlsson H. (2018). Lipoprotein modifications by gingipains of Porphyromonas gingivalis. J. Periodontal Res..

[58] Anitua E., Cerqueira A., Romero-Gavilán F., García-Arnáez I., Martinez-Ramos C., Ozturan S., Azkargorta M., Elortza F., Gurruchaga M., Goñi I. (2021). Influence of calcium ion-modified implant surfaces in protein adsorption and implant integration. Int. J. Implant. Dent..

[59] Arias-Mainer C., Romero-Gavilán F., Cerqueira A., Peñarocha-Oltra D., Bernabeu-Mira J.C., Elortza F., Azkargorta M., Gurruchaga M., Goñi I., Suay J. (2024). Combining sandblasting and pink anodisation of Ti implants as a promising method for improving fibroblast adhesion and immune response. J. Mater. Chem. B.

[60] Urieli-Shoval S., Meek R.L., Hanson R.H., Eriksen N., Benditt E.P. (1994). Human serum amyloid A genes are expressed in monocyte/macrophage cell lines. Am. J. Pathol..

[61] Steel D.M., Donoghue F.C., O’Neill R.M., Uhlar C.M., Whitehead A.S. (1996). Expression and regulation of constitutive and acute phase serum amyloid A mRNAs in hepatic and non-hepatic cell lines. Scand. J. Immunol..

[62] Jumeau C., Awad F., Assrawi E., Cobret L., Duquesnoy P., Giurgea I., Valeyre D., Grateau G., Amselem S., Bernaudin J.-F. (2019). Expression of SAA1, SAA2 and SAA4 genes in human primary monocytes and monocyte-derived macrophages. PLoS ONE.

[63] Wunderlich F., Gerovska D., Delic D., Araúzo-Bravo M.J. (2025). Protective Vaccination of Mice Against Blood-Stage Malaria Impacts Hepatic Expression of Genes Encoding Acute-Phase Proteins and IL-6 Family Members. Int. J. Mol. Sci..

[64] Meek R.L., Urieli-Shoval S., Benditt E.P. (1994). Expression of apolipoprotein serum amyloid A mRNA in human atherosclerotic lesions and cultured vascular cells: Implications for serum amyloid A function. Proc. Natl. Acad. Sci. USA.

[65] Kumon Y., Suehiro T., Hashimoto K., Sipe J.D. (2001). Dexamethasone, but not IL-1 alone, upregulates acute-phase serum amyloid A gene expression and production by cultured human aortic smooth muscle cells. Scand. J. Immunol..

[66] Kumon Y., Hosokawa T., Suehiro T., Ikeda Y., Sipe J.D., Hashimoto K. (2002). Acute-phase, but not constitutive serum amyloid A (SAA) is chemotactic for cultured human aortic smooth muscle cells. Amyloid.

[67] Liang J.S., Schreiber B.M., Salmona M., Phillip G., Gonnerman W.A., de Beer F.C., Sipe J.D. (1996). Amino terminal region of acute phase, but not constitutive, serum amyloid A (apoSAA) specifically binds and transports cholesterol into aortic smooth muscle and HepG2 cells. J. Lipid Res..

[68] Kuret T., Sodin-Šemrl S., Mrak-Poljšak K., Čučnik S., Lakota K., Erman A. (2019). Interleukin-1β Induces Intracellular Serum Amyloid A1 Expression in Human Coronary Artery Endothelial Cells and Promotes its Intercellular Exchange. Inflammation.

[69] Kovacevic A., Hammer A., Stadelmeyer E., Windischhofer W., Sundl M., Ray A., Schweighofer N., Friedl G., Windhager R., Sattler W. (2008). Expression of serum amyloid A transcripts in human bone tissues, differentiated osteoblast-like stem cells and human osteosarcoma cell lines. J. Cell Biochem..

[70] Tanaka F., Migita K., Kawabe Y., Aoyagi T., Ida H., Kawakami A., Eguchi K. (2004). Interleukin-18 induces serum amyloid A (SAA) protein production from rheumatoid synovial fibroblasts. Life Sci..

[71] Migita K., Koga T., Komori A., Torigoshi T., Maeda Y., Izumi Y., Sato J., Jiuchi Y., Miyashita T., Yamasaki S. (2011). Influence of Janus Kinase Inhibition on Interleukin 6-mediated Induction of Acute-phase Serum Amyloid A in Rheumatoid Synovium. J. Rheumatol..

[72] Sandri S., Urban Borbely A., Fernandes I., Mendes de Oliveira E., Knebel F.H., Ruano R., Zugaib M., Filippin-Monteiro F., Bevilacqua E., Campa A. (2014). Serum Amyloid A in the Placenta and Its Role in Trophoblast Invasion. PLoS ONE.

[73] Rossmann C., Hammer A., Koyani C.N., Kovacevic A., Siwetz M., Desoye G., Poehlmann T.G., Markert U.R., Huppertz B., Sattler W. (2014). Expression of serum amyloid A4 in human trophoblast-like choriocarcinoma cell lines and human first trimester/term trophoblast cells. Placenta.

[74] Ramachandran D., Dewender R.T., Schröder-Heurich B., Froböse W., Avdulahu F., Richter K., Baker V.L., Winn V.D., Pich A., von Versen-Höynck F. (2025). Exploring the plasma proteome linked to corpus luteum presence and conception mode across pregnancy stages and postpartum. J. Assist. Reprod. Genet..

[75] Urieli-Shoval S., Finci-Yeheskel Z., Eldar I., Linke R.P., Levin M., Prus D., Haimov-Kochman R. (2013). Serum amyloid A: Expression throughout human ovarian folliculogenesis and levels in follicular fluid of women undergoing controlled ovarian stimulation. J. Clin. Endocrinol. Metab..

[76] Urieli-Shoval S., Cohen P., Eisenberg S., Matzner Y. (1998). Widespread expression of serum amyloid A in histologically normal human tissues. Predominant localization to the epithelium. J. Histochem. Cytochem..

[77] Gutfeld O., Prus D., Ackerman Z., Dishon S., Linke R.P., Levin M., Urieli-Shoval S. (2006). Expression of Serum Amyloid A, in Normal, Dysplastic, and Neoplastic Human Colonic Mucosa: Implication for a Role in Colonic Tumorigenesis. J. Histochem. Cytochem..

[78] Michaeli A., Finci-Yeheskel Z., Dishon S., Linke R.P., Levin M., Urieli-Shoval S. (2008). Serum amyloid A enhances plasminogen activation: Implication for a role in colon cancer. Biochem. Biophys. Res. Commun..

[79] Calero C., Arellano E., Lopez-Villalobos J.L., Sánchez-López V., Moreno-Mata N., López-Campos J.L. (2014). Differential expression of C-reactive protein and serum amyloid A in different cell types in the lung tissue of chronic obstructive pulmonary disease patients. BMC Pulm. Med..

[80] Arellano-Orden E., Calero C., López-Ramírez C., Sánchez-López V., López-Villalobos J.L., Abad Arranz M., Blanco-Orozco A., Otero-Candelera R., López-Campos J.L. (2019). Evaluation of lung parenchyma, blood vessels, and peripheral blood lymphocytes as a potential source of acute phase reactants in patients with COPD. Int. J. Chron. Obs. Pulmon Dis..

[81] López-Campos J.L., Calero C., Rojano B., López-Porras M., Sáenz-Coronilla J., Blanco A.I., Sánchez-López V., Tobar D., Montes-Worboys A., Arellano E. (2013). C-reactive protein and serum amyloid a overexpression in lung tissues of chronic obstructive pulmonary disease patients: A case-control study. Int. J. Med. Sci..

[82] Liang J.-S., Sloane J.A., Wells J.M., Abraham C.R., Fine R.E., Sipe J.D. (1997). Evidence for local production of acute phase response apolipoprotein serum amyloid A in Alzheimer’s disease brain. Neurosci. Lett..

[83] Tucker P.C., Sack G.H. (2001). Expression of serum amyloid Agenes in mouse brain: Unprecedented response to inflammatory mediators. FASEB J..

[84] Kim Y.G., Kim M., Kang J.H., Kim H.J., Park J.W., Lee J.M., Suh J.Y., Kim J.Y., Lee J.H., Lee Y. (2016). Transcriptome sequencing of gingival biopsies from chronic periodontitis patients reveals novel gene expression and splicing patterns. Hum. Genom..

[85] Lin H.Y., Tan G.Q., Liu Y., Lin S.Q. (2019). The prognostic value of serum amyloid A in solid tumors: A meta-analysis. Cancer Cell Int..

[86] du Plessis M., Davis T., Loos B., Pretorius E., de Villiers W.J.S., Engelbrecht A.M. (2021). Molecular regulation of autophagy in a pro-inflammatory tumour microenvironment: New insight into the role of serum amyloid A. Cytokine Growth Factor. Rev..

[87] Chang Y., Liu Y., Zou Y., Ye R.D. (2025). Recent Advances in Studies of Serum Amyloid A: Implications in Inflammation, Immunity and Tumor Metastasis. Int. J. Mol. Sci..

[88] Ren Y., Wang H., Lu D., Xie X., Chen X., Peng J., Hu Q., Shi G., Liu S. (2014). Expression of serum amyloid A in uterine cervical cancer. Diagn. Pathol..

[89] Lyu P., Zhang S.D., Yuen H.F., McCrudden C.M., Wen Q., Chan K.W., Kwok H.F. (2017). Identification of TWIST-interacting genes in prostate cancer. Sci. China Life Sci..

[90] Abolbaghaei A., Turner M., Thibodeau J.-F., Holterman C.E., Kennedy C.R.J., Burger D. (2023). The Proteome of Circulating Large Extracellular Vesicles in Diabetes and Hypertension. Int. J. Mol. Sci..

[91] Vanderboom P.M., Chawla Y., Dasari S., Kapoor I., Kumar S.K., Nair K.S., Gonsalves W.I. (2024). Differences in the proteome within extracellular vesicles between premalignant and malignant plasma cell disorders. Eur. J. Haematol..

[92] Choi E.-S., Faruque H.A., Kim J.-H., Kim K.J., Choi J.E., Kim B.A., Kim B., Kim Y.J., Woo M.H., Park J.Y. (2021). CD5L as an Extracellular Vesicle-Derived Biomarker for Liquid Biopsy of Lung Cancer. Diagnostics.

[93] Milan E., Lazzari C., Anand S., Floriani I., Torri V., Sorlini C., Gregorc V., Bachi A. (2012). SAA1 is over-expressed in plasma of non small cell lung cancer patients with poor outcome after treatment with epidermal growth factor receptor tyrosine-kinase inhibitors. J. Proteom..

[94] Koc M.A., Wiles T.A., Weinhold D.C., Rightmyer S., Weaver A.L., McDowell C.T., Roder J., Asmellash S., Pestano G.A., Roder H. (2023). Molecular and translational biology of the blood-based VeriStrat^®^ proteomic test used in cancer immunotherapy treatment guidance. J. Mass. Spectrom. Adv. Clin. Lab..

[95] Chen C.L., Lin T.S., Tsai C.H., Wu C.C., Chung T., Chien K.Y., Wu M., Chang Y.S., Yu J.S., Chen Y.T. (2013). Identification of potential bladder cancer markers in urine by abundant-protein depletion coupled with quantitative proteomics. J. Proteom..

[96] Li S., Kong D., Zhang W., Li Y., Wang H., Yang R., Sun Q., Wang Z., Zhang Z. (2024). Low SAA4 gene expression is associated with advanced HCC stage and a poor prognosis. Clin. Exp. Med..

[97] Mocan L.P., Grapa C., Crăciun R., Pralea I.E., Uifălean A., Soporan A.M., Mureșan X.M., Iacobescu M., Al Hajjar N., Mihu C.M. (2024). Unveiling novel serum biomarkers in intrahepatic cholangiocarcinoma: A pilot proteomic exploration. Front. Pharmacol..

[98] Urieli-Shoval S., Finci-Yeheskel Z., Dishon S., Galinsky D., Linke R.P., Ariel I., Levin M., Ben-Shachar I., Prus D. (2010). Expression of serum amyloid a in human ovarian epithelial tumors: Implication for a role in ovarian tumorigenesis. J. Histochem. Cytochem..

[99] Li Z., Hou Y., Zhao M., Li T., Liu Y., Chang J., Ren L. (2020). Serum amyloid a, a potential biomarker both in serum and tissue, correlates with ovarian cancer progression. J. Ovarian Res..

[100] Kristjansdottir B., Partheen K., Fung E.T., Marcickiewicz J., Yip C., Brännström M., Sundfeldt K. (2012). Ovarian cyst fluid is a rich proteome resource for detection of new tumor biomarkers. Clin. Proteom..

[101] Sommella E., Capaci V., Aloisio M., Salviati E., Campiglia P., Molinario G., Licastro D., Di Lorenzo G., Romano F., Ricci G. (2022). A Label-Free Proteomic Approach for the Identification of Biomarkers in the Exosome of Endometrial Cancer Serum. Cancers.

[102] Lee J.E., Dan K., Kim H.J., Kim Y.M., Park K.H. (2022). Plasma proteomic analysis to identify potential biomarkers of histologic chorioamnionitis in women with preterm premature rupture of membranes. PLoS ONE.

[103] Urbiola-Salvador V., Jabłońska A., Miroszewska D., Kamysz W., Duzowska K., Drężek-Chyła K., Baber R., Thieme R., Gockel I., Zdrenka M. (2024). Mass Spectrometry Proteomics Characterization of Plasma Biomarkers for Colorectal Cancer Associated With Inflammation. Biomark. Insights.

[104] Pandey D., Tiwari V., Ghosh D. (2026). Small extracellular vesicles in clinical Cancer research—A quantitative proteomics perspective. J. Proteom..

[105] Yeager M.E., Colvin K.L., Everett A.D., Stenmark K.R., Ivy D.D. (2012). Plasma proteomics of differential outcome to long-term therapy in children with idiopathic pulmonary arterial hypertension. Proteom. Clin. Appl..

[106] Heywood W.E., Galimberti D., Bliss E., Sirka E., Paterson R.W., Magdalinou N.K., Carecchio M., Reid E., Heslegrave A., Fenoglio C. (2015). Identification of novel CSF biomarkers for neurodegeneration and their validation by a high-throughput multiplexed targeted proteomic assay. Mol. Neurodegener..

[107] Yang L., He L., Bu Z., Xuan C., Yu C., Wu J. (2023). Serum Protein-Based Profiles for the Diagnostic Model of Alzheimer’s Disease. Am. J. Alzheimers Dis. Other Demen.

[108] Yamada T., Miyake N., Itoh K., Igari J. (2001). Further characterization of serum amyloid A4 as a minor acute phase reactant and a possible nutritional marker. Clin. Chem. Lab. Med..

[109] Santos S.S., da Costa L., Araripe T.S.O., Reges B., Ximenes H.M.A., Moreira A. (2026). Plasma proteomic profiles reveal immune modulation by immunonutrition in GI cancer. Nutrition.

[110] Li P., Han M., Zhang R., Chen F., Li Y., Yuan J., Ma N., Li L., Wu J. (2025). Novel Biomarkers for Screening Retinal Detachment Associated with Choroidal Detachment Using DIA-MS-Based Proteomics. Curr. Eye Res..

[111] Li P., Han M., Zhang R., Chen F., Li Y., Yuan J., Ma N., Li L., Wu J. (2025). Efficacy of Glucocorticoids in the Treatment of Retinal Detachment with Choroidal Detachment: Analysis by Proteomics. Proteom. Clin. Appl..

[112] Liu Q., Sun S., Yang Z., Shao Y., Li X. (2023). Serum Amyloid A 4 as a Common Marker of Persistent Inflammation in Patients with Neovascular Age-Related Macular Degeneration and Polypoidal Choroidal Vasculopathy. J. Inflamm. Res..

[113] Ray S., Kumar V., Bhave A., Singh V., Gogtay N.J., Thatte U.M., Talukdar A., Kochar S.K., Patankar S., Srivastava S. (2015). Proteomic analysis of Plasmodium falciparum induced alterations in humans from different endemic regions of India to decipher malaria pathogenesis and identify surrogate markers of severity. J. Proteom..

[114] Upur H., Chen Y., Kamilijiang M., Deng W., Sulaiman X., Aizezi R., Wu X., Tulake W., Abudula A. (2015). Identification of plasma protein markers common to patients with malignant tumour and Abnormal Savda in Uighur medicine: A prospective clinical study. BMC Complement. Altern. Med..

[115] De Bock M., Beguin Y., Leprince P., Willems E., Baron F., Deroyer C., Seidel L., Cavalier E., de Seny D., Malaise M. (2014). Comprehensive plasma profiling for the characterization of graft-versus-host disease biomarkers. Talanta.

[116] Vidova V., Stuchlikova E., Vrbova M., Almasi M., Klanova J., Thon V., Spacil Z. (2019). Multiplex Assay for Quantification of Acute Phase Proteins and Immunoglobulin A in Dried Blood Spots. J. Proteome Res..

[117] Kumon Y., Loose L.D., Birbara C.A., Sipe J.D. (1997). Rheumatoid arthritis exhibits reduced acute phase and enhanced constitutive serum amyloid A protein in synovial fluid relative to serum. A comparison with C-reactive protein. J. Rheumatol..

[118] Lee J.S., Chapman M.J., Piraino P., Lamerz J., Schindler T., Cutler P., Dernick G. (2016). Remodeling of plasma lipoproteins in patients with rheumatoid arthritis: Interleukin-6 receptor-alpha inhibition with tocilizumab. Proteom. Clin. Appl..

[119] Seok A., Lee H.-J., Lee S., Lee J., Mun S., Park A., Chun Y.-T., Lee J.-H., Lim H.-J., Kang H.-G. (2017). Identification and Validation of SAA4 as a Rheumatoid Arthritis Prescreening Marker by Liquid Chromatography Tandem-mass Spectrometry. Molecules.

[120] Mun S., Lee J., Lim M.-K., Lee Y.-R., Ihm C., Lee S.H., Kang H.-G. (2018). Development of a Novel Diagnostic Biomarker Set for Rheumatoid Arthritis Using a Proteomics Approach. BioMed Res. Int..

[121] Mun S., Lee J., Park A., Kim H.J., Lee Y.J., Son H., Shin M., Lim M.K., Kang H.G. (2019). Proteomics Approach for the Discovery of Rheumatoid Arthritis Biomarkers Using Mass Spectrometry. Int. J. Mol. Sci..

[122] Mun S., Lee J., Park M., Shin J., Lim M.K., Kang H.G. (2021). Serum biomarker panel for the diagnosis of rheumatoid arthritis. Arthritis Res. Ther..

[123] Cuesta-López L., Escudero-Contreras A., Hanaee Y., Pérez-Sánchez C., Ruiz-Ponce M., Martínez-Moreno J.M., Pérez-Pampin E., González A., Plasencia-Rodriguez C., Martínez-Feito A. (2024). Exploring candidate biomarkers for rheumatoid arthritis through cardiovascular and cardiometabolic serum proteome profiling. Front. Immunol..

[124] Li J., Liu H., Dai B., Fan Z., Wang Q., Liu H., Wei T., Bai B., Fu L. (2022). SAA4 is a Diagnostic Marker to Enhance Detection Forrheumatoid Arthritis Combined with Anti-CCP. Res. Sq..

[125] Decker R., Andersson B., Nierop A.F.M., Bosaeus I., Dahlgren J., Albertsson-Wikland K., Hellgren G. (2013). Protein markers predict body composition during growth hormone treatment in short prepubertal children. Clin. Endocrinol..

[126] Andersson B., Decker R., Nierop A.F., Bosaeus I., Albertsson-Wikland K., Hellgren G. (2011). Protein profiling identified dissociations between growth hormone-mediated longitudinal growth and bone mineralization in short prepubertal children. J. Proteom..

[127] Andersson B., Hellgren G., Nierop A.F.M., Hochberg Z., Albertsson-Wikland K. (2009). Proteins related to lipoprotein profile were identified using a pharmaco-proteomic approach as markers for growth response to growth hormone (GH) treatment in short prepubertal children. Proteome Sci..

[128] Ijsselstijn L., Papma J.M., Dekker L.J.M., Calame W., Stingl C., Koudstaal P.J., Prins N.D., Sillevis Smitt P.A.E., Luider T.M. (2013). Serum proteomics in amnestic mild cognitive impairment. Proteomics.

[129] Lee S., Mun S., Joo E.-J., Yun Y., Kang H.-G., Lee J. (2024). Serum proteomic changes related to residual impairment in remittent depression are associated with immune and inflammatory processes. Sci. Rep..

[130] Smagin D.A., Bezryadnov D.V., Zavialova M.G., Abramova A.Y., Pertsov S.S., Kudryavtseva N.N. (2024). Blood Plasma Markers in Depressed Mice under Chronic Social Defeat Stress. Biomedicines.

[131] Bellei E., Rustichelli C., Bergamini S., Monari E., Baraldi C., Lo Castro F., Tomasi A., Ferrari A. (2020). Proteomic serum profile in menstrual-related and post menopause migraine. J. Pharm. Biomed. Anal..

[132] Connolly B., McCreight L., Slieker R.C., Bedair K.F., Donnelly L., de Klerk J.A., Beulens J.W.J., Elders P.J.M., Bergström G., Hong M.G. (2025). The influence of metformin treatment on the circulating proteome. EBioMedicine.

[133] Meng H., Ruan J., Chen Y., Yan Z., Shi K., Li X., Yang P., Meng F. (2021). Investigation of Specific Proteins Related to Different Types of Coronary Atherosclerosis. Front. Cardiovasc. Med..

[134] Sharma N.K., Ferreira B.L., Tashima A.K., Brunialti M.K.C., Torquato R.J.S., Bafi A., Assuncao M., Azevedo L.C.P., Salomao R. (2019). Lipid metabolism impairment in patients with sepsis secondary to hospital acquired pneumonia, a proteomic analysis. Clin. Proteom..

[135] Sarkar S., Elliott E.C., Henry H.R., Ludovico I.D., Melchior J.T., Frazer-Abel A., Webb-Robertson B.J., Davidson W.S., Holers V.M., Rewers M.J. (2023). Systematic review of type 1 diabetes biomarkers reveals regulation in circulating proteins related to complement, lipid metabolism, and immune response. Clin. Proteom..

[136] Bril F., Pearce R.W., Collier T.S., McPhaul M.J. (2022). Differences in HDL-Bound Apolipoproteins in Patients With Advanced Liver Fibrosis Due to Nonalcoholic Fatty Liver Disease. J. Clin. Endocrinol. Metab..

[137] Captur G., Doykov I., Chung S.C., Field E., Barnes A., Zhang E., Heenan I., Norrish G., Moon J.C., Elliott P.M. (2024). Novel Multiplexed Plasma Biomarker Panel Has Diagnostic and Prognostic Potential in Children With Hypertrophic Cardiomyopathy. Circ. Genom. Precis. Med..

[138] Li X., Luo T., Yan H., Xie L., Yang Y., Gong L., Tang Z., Tang M., Zhang X., Huang J. (2023). Proteomic Analysis of Pediatric Hemophagocytic Lymphohistiocytosis: A Comparative Study with Healthy Controls, Sepsis, Critical Ill, and Active Epstein-Barr virus Infection to Identify Altered Pathways and Candidate Biomarkers. J. Clin. Immunol..

[139] Da’dara A.A., Siddons G., Icaza M., Wang Q., Skelly P.J. (2017). How schistosomes alter the human serum proteome. Mol. Biochem. Parasitol..

[140] Yao X., Qiao B., Shan F., Zhang Q., Song Y., Song J., Wang Y. (2025). Elevated Serum Amyloid A2 and A4 in Patients With Guillain-Barré Syndrome. J. Clin. Neurol..

[141] Fernández J.A., Deguchi H., Elias D.J., Griffin J.H. (2020). Serum amyloid A4 is a procoagulant apolipoprotein that it is elevated in venous thrombosis patients. Res. Pract. Thromb. Haemost..

[142] Merritt E.K., Nieman D.C., Toone B.R., Groen A., Pugachev A. (2019). Proteomic Markers of Non-functional Overreaching During the Race Across America (RAAM): A Case Study. Front. Physiol..

[143] Choi S., Park Y.E., Cheon E.J., Kim K.Y., Kim M., Ann S.J., Noh H.M., Lee J., Lee C.J., Lee S.T. (2020). Novel Associations between Related Proteins and Cellular Effects of High-Density Lipoprotein. Korean Circ. J..

[144] Marsche G., Frank S., Raynes J.G., Kozarsky K.F., Sattler W., Malle E. (2007). The lipidation status of acute-phase protein serum amyloid A determines cholesterol mobilization via scavenger receptor class B, type I. Biochem. J..

[145] Stonik J.A., Remaley A.T., Demosky S.J., Neufeld E.B., Bocharov A., Brewer H.B. (2004). Serum amyloid A promotes ABCA1-dependent and ABCA1-independent lipid efflux from cells. Biochem. Biophys. Res. Commun..

[146] Freeman W.M., Vanguilder H.D., Guidone E., Krystal J.H., Grant K.A., Vrana K.E. (2011). Plasma proteomic alterations in non-human primates and humans after chronic alcohol self-administration. Int. J. Neuropsychopharmacol..

[147] Coppola D., Balducci L., Chen D.T., Loboda A., Nebozhyn M., Staller A., Fulp W.J., Dalton W., Yeatman T., Brem S. (2014). Senescence-associated-gene signature identifies genes linked to age, prognosis, and progression of human gliomas. J. Geriatr. Oncol..

[148] Irvine K.M., Skoien R., Bokil N.J., Melino M., Thomas G.P., Loo D., Gabrielli B., Hill M.M., Sweet M.J., Clouston A.D. (2014). Senescent human hepatocytes express a unique secretory phenotype and promote macrophage migration. World J. Gastroenterol..

[149] Orwoll E.S., Wiedrick J., Jacobs J., Baker E.S., Piehowski P., Petyuk V., Gao Y., Shi T., Smith R.D., Bauer D.C. (2018). High-throughput serum proteomics for the identification of protein biomarkers of mortality in older men. Aging Cell.

[150] Hirakura Y., Carreras I., Sipe J.D., Kagan B.L. (2002). Channel formation by serum amyloid A: A potential mechanism for amyloid pathogenesis and host defense. Amyloid.

[151] Hrzenjak A., Artl A., Knipping G., Kostner G., Sattler W., Malle E. (2001). Silent mutations in secondary Shine–Dalgarno sequences in the cDNA of human serum amyloid A4 promotes expression of recombinant protein in Escherichia coli. Protein Eng..

[152] Li X., Shen J., Chen X., Chen L., Wan S., Qiu X., Chen K., Chen C., Tan H. (2022). Humanization of Yeasts for Glycan-Type End-Products. Front. Microbiol..

[153] Bretthauer R.K. (2003). Genetic engineering of Pichia pastoris to humanize N-glycosylation of proteins. Trends Biotechnol..

[154] Hamilton S.R., Bobrowicz P., Bobrowicz B., Davidson R.C., Li H., Mitchell T., Nett J.H., Rausch S., Stadheim T.A., Wischnewski H. (2003). Production of Complex Human Glycoproteins in Yeast. Science.

[155] Abouelasrar Salama S., De Bondt M., Berghmans N., Gouwy M., de Oliveira V.L.S., Oliveira S.C., Amaral F.A., Proost P., Van Damme J., Struyf S. (2020). Biological Characterization of Commercial Recombinantly Expressed Immunomodulating Proteins Contaminated with Bacterial Products in the Year 2020: The SAA3 Case. Mediat. Inflamm..

[156] Van Damme J., Struyf S., Proost P., Opdenakker G., Gouwy M. (2025). Functional Interactions Between Recombinant Serum Amyloid A1 (SAA1) and Chemokines in Leukocyte Recruitment. Int. J. Mol. Sci..

[157] Mohanty T., Miličević K., Göthert H., Tillmann A., Padra M., Papareddy P., Herwald H. (2025). Balancing inflammation: The specific roles of serum amyloid A proteins in sterile and infectious diseases. Front. Immunol..

[158] Meek R.L., Benditt E.P. (1989). Rat tissues express serum amyloid A protein-related mRNAs. Proc. Natl. Acad. Sci. USA.

[159] Baltz M.L., Rowe I.F., Caspi D., Turnell W.G., Pepys M.B. (1987). Acute-phase high-density lipoprotein in the rat does not contain serum amyloid A protein. Biochem. J..

[160] Rossmann C., Windpassinger C., Brunner D., Kovacevic A., Schweighofer N., Malli R., Schuligoi R., Prokesch A., Kluve-Beckerman B., Graier W.F. (2014). Characterization of rat serum amyloid A4 (SAA4): A novel member of the SAA superfamily. Biochem. Biophys. Res. Commun..

[161] Li X., Liao W.S.L. (1992). Cooperative effects of C/EBP-like and NFxB-like binding sites on rat serum amyloid A1 gene expression in liver cells. Nucleic Acids Res..

[162] Inoue Y., Inoue J., Lambert G., Yim S.H., Gonzalez F.J. (2004). Disruption of hepatic C/EBPalpha results in impaired glucose tolerance and age-dependent hepatosteatosis. J. Biol. Chem..

[163] Murphy C.L., Wang S., Kestler D.P., Stevens F.A., Weiss D.T., Solomon A. (2009). AA amyloidosis associated with a mutated serum amyloid A4 protein. Amyloid.

[164] Yamada T., Kluve-Beckerman B., Liepnieks J.J., Benson M.D. (1994). Fibril formation from recombinant human serum amyloid A. Biochim. Biophys. Acta (BBA) Mol. Basis Dis..

[165] Dasari S., Theis J.D., Vrana J.A., Zenka R.M., Zimmermann M.T., Kocher J.-P.A., Highsmith W.E., Kurtin P.J., Dogan A. (2014). Clinical Proteome Informatics Workbench Detects Pathogenic Mutations in Hereditary Amyloidoses. J. Proteome Res..

[166] Olsen H.G., Skovgaard K., Nielsen O.L., Leifsson P.S., Jensen H.E., Iburg T., Heegaard P.M.H. (2013). Organization and Biology of the Porcine Serum Amyloid A (SAA) Gene Cluster: Isoform Specific Responses to Bacterial Infection. PLoS ONE.

[167] Sallustio F., Stasi A., Curci C., Divella C., Picerno A., Franzin R., De Palma G., Rutigliano M., Lucarelli G., Battaglia M. (2019). Renal progenitor cells revert LPS-induced endothelial-to-mesenchymal transition by secreting CXCL6, SAA4, and BPIFA2 antiseptic peptides. FASEB J..

[168] Julian C.G., Subudhi A.W., Hill R.C., Wilson M.J., Dimmen A.C., Hansen K.C., Roach R.C. (2014). Exploratory proteomic analysis of hypobaric hypoxia and acute mountain sickness in humans. J. Appl. Physiol..

[169] González-Buenfil R., Vieyra-Sánchez S., Quinto-Cortés C.D., Oppenheimer S.J., Pomat W., Laman M., Cervantes-Hernández M.C., Barberena-Jonas C., Auckland K., Allen A. (2024). Genetic Signatures of Positive Selection in Human Populations Adapted to High Altitude in Papua New Guinea. Genome Biol. Evol..

[170] Malle E., Leonhard B., Knipping G., Sattler W. (1999). Effects of Cytokines, Butyrate and Dexamethasone on Serum Amyloid A and Apolipoprotein A-I Synthesis in Human HUH-7 Hepatoma Cells. Scand. J. Immunol..

[171] Cavalcante J.S., Borges da Silva W.R.G., de Oliveira L.A., Brito I.M.C., Muller K.S., Vidal I.S.J., dos Santos L.D., Jorge R.J.B., Almeida C., de Lima Bicho C. (2022). Blood plasma proteome alteration after local tissue damage induced by Bothrops erythromelas snake venom in mice. J. Proteom..

